# The dentition of the Late Jurassic dwarf sauropod *Europasaurus holgeri* from northern Germany: ontogeny, function, and implications for a rhamphotheca-like structure in Sauropoda

**DOI:** 10.7717/peerj.17764

**Published:** 2024-08-13

**Authors:** Verena Régent, Kayleigh Wiersma-Weyand, Oliver Wings, Nils Knötschke, P. Martin Sander

**Affiliations:** 1 Abteilung Paläontologie, Institut für Geowissenschaften, Universität Bonn, Bonn, Germany; 2 Zentralmagazin Naturwissenschaftlicher Sammlungen, Martin-Luther-Universität Halle-Wittenberg, Halle, Germany; 3 Museum für Naturkunde Berlin, Berlin, Germany; 4 Naturkundemuseum Bamberg, Bamberg, Bavaria, Germany; 5 Dinosaurier-Freilichtmuseum Münchehagen, Rehburg-Loccum, Germany; 6 The Dinosaur Institute, Natural History Museum of Los Angeles County, Los Angeles, California, United States of America

**Keywords:** Europasaurus, Dentition, Sauropoda, Jurassic, Tooth replacement, Tooth wear, Isolated tooth row, Rhamphotheca

## Abstract

The basal macronarian sauropod *Europasaurus holgeri* is known only from the Late Jurassic of the Langenberg Quarry near Goslar, Lower Saxony, Germany. *Europasaurus* has been identified as an insular dwarf and shows a clear resemblance to *Camarasaurus* and *Giraffatitan*. This study provides a detailed description of the dentition of *Europasaurus* based on an array of fossils outstanding in their abundance, variety of preservation, and ontogenetic range. Dental morphology for the replacement and functional dentitions, the tooth replacement pattern, and implications for food intake are described for the *Europasaurus* dentition, which is characterized by broad-crowned teeth. Characteristic features for *Europasaurus* are the presence of denticles on replacement teeth, the wrinkled enamel, and large wear facets both on the apex and on the carinae of the tooth crowns. The partially articulated skull SNHM-2207-R and isolated tooth rows DfmMh/FV 580.1 and DfmMh/FV 896.7 suggest the presence of strong connective tissue partially covering the teeth. This connective tissue would have provided stability and protection for the teeth. Evidence for this connective tissue include exposed tooth necks, *in-situ* teeth with strongly resorbed roots which no longer would have been connected to the jaw bone, and wrinkled enamel and its surface pattern. The same features can be observed in other sauropod taxa as well. We therefore suggest that eusauropods in general possessed this connective tissue structure, which may be an autapomorphy of the group. Possibly, this hypothetical structure is homologous to the rhamphotheca in birds and some non-avian theropods, which, however rarely, show such a close integration of keratinous tissue and teeth that we hypothesize here.

## Introduction

Sauropod dinosaurs were the largest terrestrial vertebrates ever and are characterized by gigantic bodies with pillar-like extremities, exceptionally elongated necks and tails, and comparably minute skulls (Upchurch, Barrett & Dodson, 2004; Fastovsky & Weishampel, 2005; Sander et al., 2011). Sauropod dinosaur remains are found worldwide, yet fossils from the Langenberg Quarry near Goslar, Lower Saxony, Germany, are among the most important. Even the largest elements do not exceed the juvenile size of other sauropods, leading to the initial hypothesis that the Langenberg assemblage contains only juveniles. However, bone histology shows that the remains belong to a dwarf sauropod which was recognized as a new taxon, *Europasaurus holgeri* (Sander et al., 2006), so far only recorded from the Langenberg Quarry. All parts of the skeleton have been described in detail from a comprehensive growth series, and two morphs have been recognized (Carballido & Sander, 2014; Marpmann et al., 2015; Carballido et al., 2019). Adult *Europasaurus* reached a body length of only about 6–8 m compared to well over 20 m in its closest relatives. *Europasaurus* represented the first unequivocal case of island dwarfism in dinosaurs (Sander et al., 2006), although a Late Cretaceous fauna of unusually small dinosaurs from Romania has recently been confirmed as island dwarfs too (Weishampel, Grigorescu & Norman, 1991; Weishampel, Norman & Grigorescu, 1993; Benton et al., 2010; Stein et al., 2010).

Apart from some early and influential papers (Janensch, 1935–1936; Nowinski, 1971; Barrett & Upchurch, 1994; Calvo, 1994; Fiorillo, 1998), the dentition and feeding strategies of sauropods had not figured prominently in the “dinosaur renaissance” (but see bibliography in Whitlock, 2011). However, the last two decades have seen a huge interest in sauropod dental morphology, tooth replacement, and evolution of their dentition (Christiansen, 2000; Chatterjee & Zheng, 2002, 2005; Sereno & Wilson, 2005; Barrett, 2006; Sereno et al., 2007; Chure et al., 2010; García & Cerda, 2010a, 2010b; Young et al., 2012; D’Emic et al., 2013; Díez Díaz, Tortosa & Le Loeuff, 2013; Díez Díaz, Ortega & Sanz, 2014; Kosch et al., 2014; Holwerda, Pol & Rauhut, 2015; Wings et al., 2015; Barrett et al., 2016; García & Zurriaguz, 2016; Mocho et al., 2016; Wilson et al., 2016; Averianov & Sues, 2017; Carballido et al., 2017; Mocho et al., 2017; Ősi, Csiki-Sava & Prondvai, 2017; Wiersma & Sander, 2017; Holwerda et al., 2018; McHugh, 2018; Moore et al., 2018; Tschopp, Mateus & Norell, 2018; Woodruff et al., 2018; D’Emic & Carrano, 2020; Bindellini & Dal Sasso, 2021; Chang et al., 2021; Price & Whitlock, 2022; Peterson et al., 2022; Torcida Fernández-Baldor et al., 2023). Our study presents a detailed morphological description of the dentition of *Europasaurus holgeri*. Among sauropods, this taxon is represented by one of the richest collections of jaw and dental material, uniquely including an extensive growth series spanning a wide size range. The material consists of a partial skull, numerous dentigerous bones, isolated tooth rows, and isolated teeth. Our focus is on tooth replacement, functional and ontogenetic patterns, soft part reconstruction, and their significance for the biology and behavior of *Europasaurus*. In particular, we explore the meaning of the peculiar and unique isolated tooth rows—abbreviated ITRs in the following—of *Europasaurus* and several other eusauropods.

### Locality and taphonomy of *Europasaurus* jaw and dental remains

The Langenberg Quarry is comprised of late Oxfordian to late Kimmeridgian limestone beds that crop out at the northern rim of the Harz Mountains near Goslar, Germany (Lotze, 1968; Pape, 1970; Fischer, 1991). Despite their deposition in a marine environment, some beds contain terrestrial vertebrate remains including lizards, atoposaurid crocodyliforms, pterosaurs, sauropod dinosaur*s*, several taxa of theropod dinosaurs, and multituberculate mammals (*e.g*., Fastnacht, 2005; Sander et al., 2006; Wings & Sander, 2012; Richter et al., 2013; Carballido & Sander, 2014; Gerke & Wings, 2014; Marpmann et al., 2015; Lallensack et al., 2015; Wings, 2015; Schwarz, Raddatz & Wings, 2017; Martin, 2018). *Europasaurus* constitutes more than 99% of the dinosaur material discovered in bed 83 of Pape (1970) of the Langenberg section, and the taxon is only known from this bed (Scheil & Sander, 2017). The abundant and exquisitely three-dimensionally preserved material varies from isolated bones to associated partial skeletons of individuals widely differing in ontogenetic stage. So far, no fully articulated material has been reported (see Carballido & Sander, 2014; Marpmann et al., 2015; Scheil & Sander, 2017; Carballido et al., 2019).

### Review of the evolution of the sauropodomorph dentition

Within Sauropodomorpha (Upchurch, 1995; Sander, 1997; Barrett & Upchurch, 2005, 2007), the simplest teeth are found in non-sauropod sauropodomorphs, which are blade-like and flattened labiolingually with an expanded crown (Galton, 1985; Sander, 1997; Galton & Upchurch, 2004), with coarse denticles along the carinae (Galton, 1985; Sander, 1997). Both, the labial side and the lingual side are convex, and the lingual side shows a pronounced ridge from the base of the crown over its entire length.

Non-neosauropod sauropods have more complex teeth of a diversity of crown shapes and often show extensive enamel wrinkling (see below). Within the more derived, *i.e*., neosauropod, sauropods, two main tooth morphotypes, broad-crowned and narrow-crowned, can be distinguished (Huene, 1926; Janensch, 1929, 1935–1936; Upchurch, 1995, 1998; Sander, 1997; Wilson & Sereno, 1998; Upchurch & Barrett, 2000; Chatterjee & Zheng, 2002; Wilson, 2002; Upchurch, Barrett & Dodson, 2004; Barrett & Upchurch, 2005; Chure et al., 2010; Sander et al., 2011).

Broad-crowned teeth are spoon- or shovel-shaped and are characteristic of basal Eusauropoda and basal Macronaria (Janensch, 1935–1936; Upchurch, 1995, 1998; Sander, 1997; Wilson & Sereno, 1998; Sanz et al., 1999; Upchurch & Barrett, 2000; Chatterjee & Zheng, 2002; Wilson, 2002; Upchurch, Barrett & Dodson, 2004; Barrett & Upchurch, 2005, 2007; Chure et al., 2010; Sander et al., 2011; Wiersma & Sander, 2017). The other morphotype, narrow-crowned teeth, also described as pencil- or peg-shaped, are generally less robust than broad-crowned teeth (Sander, 1997; Upchurch, 1998; Upchurch & Barrett, 2000; Barrett & Upchurch, 2005). They evolved convergently at least twice within the Diplodocoidea and derived Titanosauriformes (Calvo & Salgado, 1995; Upchurch, 1995; 1998; Salgado, Coria & Calvo, 1997; Sander, 1997; Wilson & Sereno, 1998; Sanz et al., 1999; Christiansen, 2000; Upchurch & Barrett, 2000; Curry Rogers & Forster, 2001; Wilson, 2002; Upchurch, Barrett & Dodson, 2004; Barrett & Upchurch, 2005; Chure et al., 2010; Sander et al., 2010, 2011; Wiersma & Sander, 2017).

Alternative, more elaborate classifications than broad-crowned and narrow-crowned have also been used in sauropodomorphs, such as spoon-like teeth, peg-like teeth, cone-chisel, and chisel- like teeth (Calvo, 1994), vulcanodontid teeth (leaf-shaped), spatulate teeth (in *Euhelopus*, *Shunosaurus*, Mamenchisauridae), mesiodistally expanded spatulate teeth (in *Camarasaurus*), cone-chisel like teeth (in Brachiosauridae), slender cone-chisel like teeth (in Titanosauridae), peg-like teeth (in Nemegtosauridae and Diplodocoidea) (Upchurch & Barrett, 2000), leaf-like teeth (the non-sauropod *Anchisaurus*), spoon-shaped teeth (in *Kotasaurus*, *Barapasaurus*), spatulate teeth (in Shunosauridae, Mamenchisauridae, Euhelopodidae, Brachiosauridae, *Patagosaurus*), teeth with a nearly circular diameter (in Diplodocoidea, Titanosauridae) (Stevens & Parrish, 2005).

Sauropod teeth generally possess a distinctly wrinkled enamel surface, and different patterns of wrinkling have been described with the aim of distinguishing taxa and clades (*e.g*., Holwerda, Pol & Rauhut, 2015; Mocho et al., 2017; Holwerda et al., 2018; Bindellini & Dal Sasso, 2021). Wrinkled enamel was recognized as a synapomorphy of Eusauropoda (Wilson & Sereno, 1998; Allain & Aquesbi, 2008; Holwerda, Pol & Rauhut, 2015; Carballido et al., 2017; Wiersma & Sander, 2017). This feature, together with the ITRs, may indicate the presence of soft tissue holding the teeth in place after death (Wiersma & Sander, 2017).

Clearly, understanding tooth morphology and jaw morphology, and reconstructing soft part anatomy are of crucial paleobiological importance for understanding sauropods as living animals (Sander & Clauss, 2008; Klein et al., 2011; Sander et al., 2011). Sauropods are unique in the ratio of skull mass to total body mass, and their entire energy uptake must have occurred through those relatively minute skulls. Extreme adaptations thus should not come as a surprise, and major and detailed efforts in understanding their jaw function and feeding adaptations are wholly justified (*e.g*., Whitlock, 2017).

## Materials and Methods

### Material

The majority of the *Europasaurus* bones and teeth, including a well preserved partial skull (Laven, 2001; Marpmann et al., 2015) was discovered between 1998 and 2002. Because the locality is an active quarry and the strata are steeply inclined (slightly overturned), only limited systematic excavations were possible. Instead, rock stockpiles were screened after every blasting, and fossil-bearing blocks were collected. Currently, more than 1,300 bones and teeth of *Europasaurus*, belonging to at least 21 individuals of different sizes and ages have been excavated (Scheil & Sander, 2017). The fossils are accessioned to the collections of the Verein zur Förderung der niedersächsischen Paläontologie (FV) which currently is housed at the DfmMh, resulting in the collections acronym DfmMh/FV.

Our study is based on a rich and diverse sample. It consists of a variety of well-preserved isolated tooth-bearing jaw elements of different ontogenetic stages (Marpmann et al., 2015), two ITRs (DfmMh/FV 580.1 and DfmMh/FV 896.7), a partially articulated skull (original SNHM-2207-R and its cast from an earlier preparation stage, NLMH 105.996), and 90 isolated teeth recovered from the matrix of the fossiliferous blocks (see above on collection history). All isolated teeth are well-preserved and lack indications of post-mortem transport. Most teeth were found isolated, in the micritic limestone matrix, only a few were situated adjacent to bones. Two more *Europasaurus* ITRs have been found but could not be studied: one ITR in the DfmMh/FV collection had been lost in a fire, and another ITR is part of a private collection (Marcus Schipplick, Braunschweig).

The isolated tooth-bearing jaw elements investigated are a total of 13 dentaries (DfmMh/FV 033, DfmMh/FV 034, DfmMh/FV 059, DfmMh/FV 092, DfmMh/FV 093, DfmMh/FV 094, DfmMh/FV 290, DfmMh/FV 291.11-holotype, DfmMh/FV 501, DfmMh/FV 653, DfmMh/FV 654, DfmMh/FV 834.7, and DfmMh/FP 1058.14), five premaxillae (DfmMh/FV 32, DfmMh/FV 61, DfmMh/FV 652.2, DfmMh/FV 982, and DfmMh/FV 291.18-holotype), but only one maxilla (DfmMh/FV 291.17-holotype) (see also Marpmann et al., 2015). Note that the isolated jaw bones consistently lack any functional teeth which apparently were prone to postmortem loss from their respective alveoli, being preserved individually or as the ITRs. The partially articulated skull (SNHM-2207-R, cast NLMH 106.996) preserves the right and the left dentary, a part of the left maxilla, and a large part of the dentition. This skull also documents the seemingly loose implantation of the teeth in the jaws and their rapid post-mortem loss from the alveoli. Thus, we investigated dental wear in isolated teeth as well. We note that it was not possible to assign the material described in this study to the two size classes apparent in the skull (Marpmann et al., 2015) and axial skeleton (Carballido & Sander, 2014) of *Europasaurus holgeri*.

For comparison, the following material was studied first hand: *Giraffatitan* specimens from the Tendaguru Formation (MFN t1, now MB.R.2223; S66, now MB.R.2180; S116, now MB.R.2181; and WJ 4170, now MB.R. 2390), *Camarasaurus* sp. specimen SMA 0002 ‘ET’ (Wiersma & Sander, 2017), holotype specimen of *Kaatedocus siberi* SMA 0004 (Tschopp, Mateus & Benson, 2015), and *Galeamopus* sp. specimen SMA 0011 ‘Max’ (Tschopp, Mateus & Benson, 2015) from the Late Jurassic Morrison Formation.

### Methods

Morphological examinations were performed macroscopically by sight and microscopically under a stereo microscope. An electronic caliper (measurement error +/− 0.5 mm) was used, and the total preserved length as well as the crown length was measured for all teeth. The length of the crown was measured along the longitudinal axis from the crown tip to the base of the enamel cap. The width of the crown was measured along the widest mesiodistal expansion. These measurements were used to calculate the slenderness index (SI), which represents the ratio between crown height and crown width (Barrett & Upchurch, 2005). We follow the usage of Upchurch (1998), Upchurch & Barrett (2005), and Chure et al. (2010) in that broad-crowned teeth have an SI of ≤4.0, and narrow-crowned teeth have an SI of ≥4.0. We note that other cut-offs have been used (*e.g*., D’Emic, 2012; Mannion et al., 2013).

The terminologies used in dentistry, in the descriptions of reptile teeth (Edmund, 1969; Smith & Dodson, 2003), and in the descriptions of sauropod dentitions (Wiersma & Sander, 2017) were used in this study.

**Figure 1:**
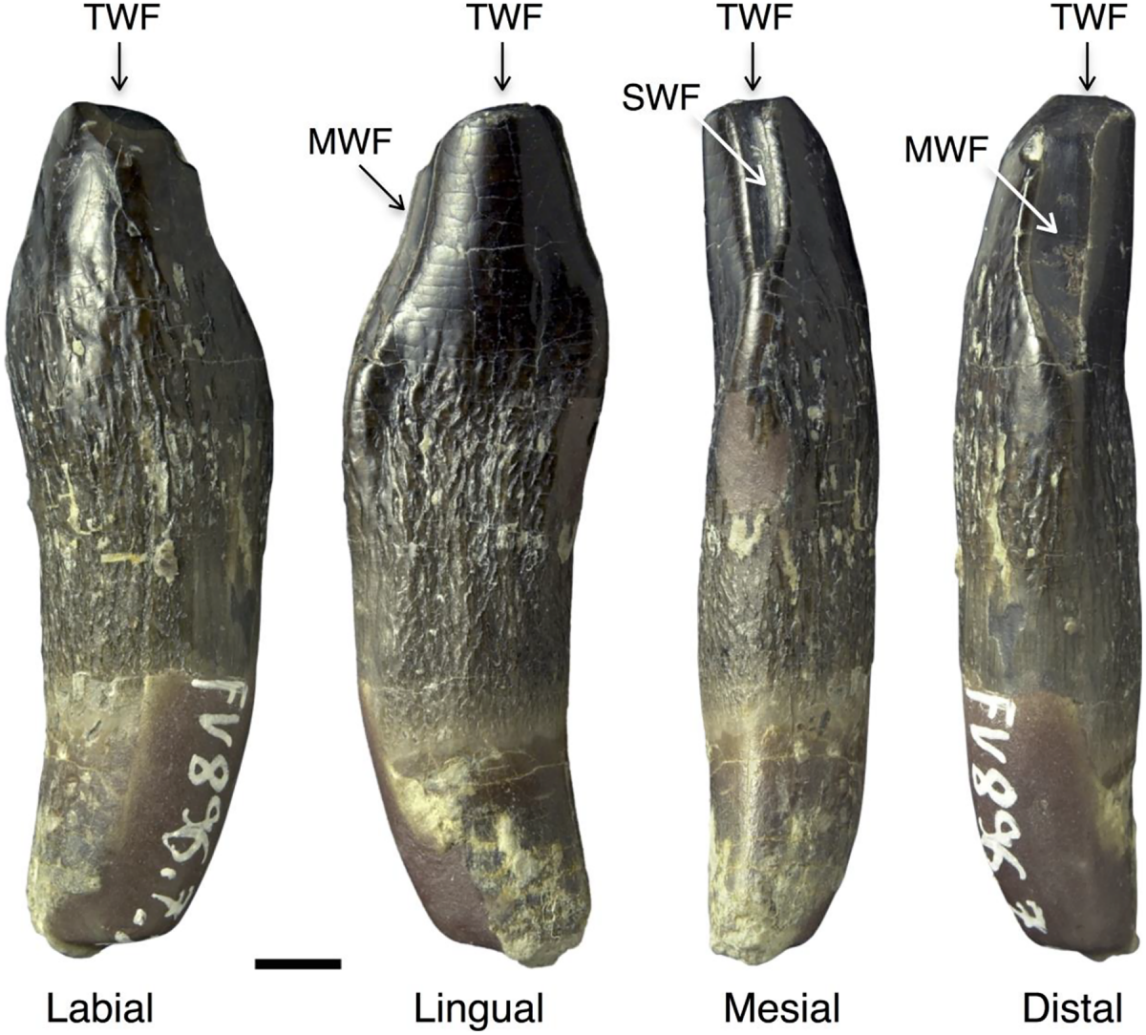
Wear facets in the teeth of *Europasaurus holgeri*. The facets are illustrated based on the first left dentary tooth FV 896.7.2 which is part of isolated tooth row DfmMh/FV 896.7 (see also Fig. 7). Tooth FV 896.7.2 was separated from the matrix during preparation to reveal its morphology from all directions. Note that the position of the main and side wear facets are switched in upper and lower teeth. Scale = 3 mm. Wear facet abbreviations: MWF, main wear facet; SWF, side wear facet; TWF, terminal wear facet.

The wear stages of the teeth were described according to Janensch (1935–1936), Chatterjee & Zheng (2002, 2005), and Wiersma & Sander (2017). Three wear facets were described by these authors and are used in this study (Fig. 1). First, the terminal (or occlusal) wear facet occurs only at the tip of the crown, possesses a round to oval shape, and is steeply inclined. We prefer “terminal” over “occlusal” because of the purely descriptive nature of the former term. The second wear facet is the elongated main wear facet that occurs on one of the carinae of the crown (lower jaw: distal; upper jaw: mesial, Fig. 1). This wear facet is larger than the terminal wear facet. The side wear facet (Fig. 1) is similar to the main wear facet, but is located on the opposite carina of the crown (lower jaw: mesial; upper jaw: distal), and it is usually not as well developed as the main wear facet (Fig. 1).

**Figure 7:**
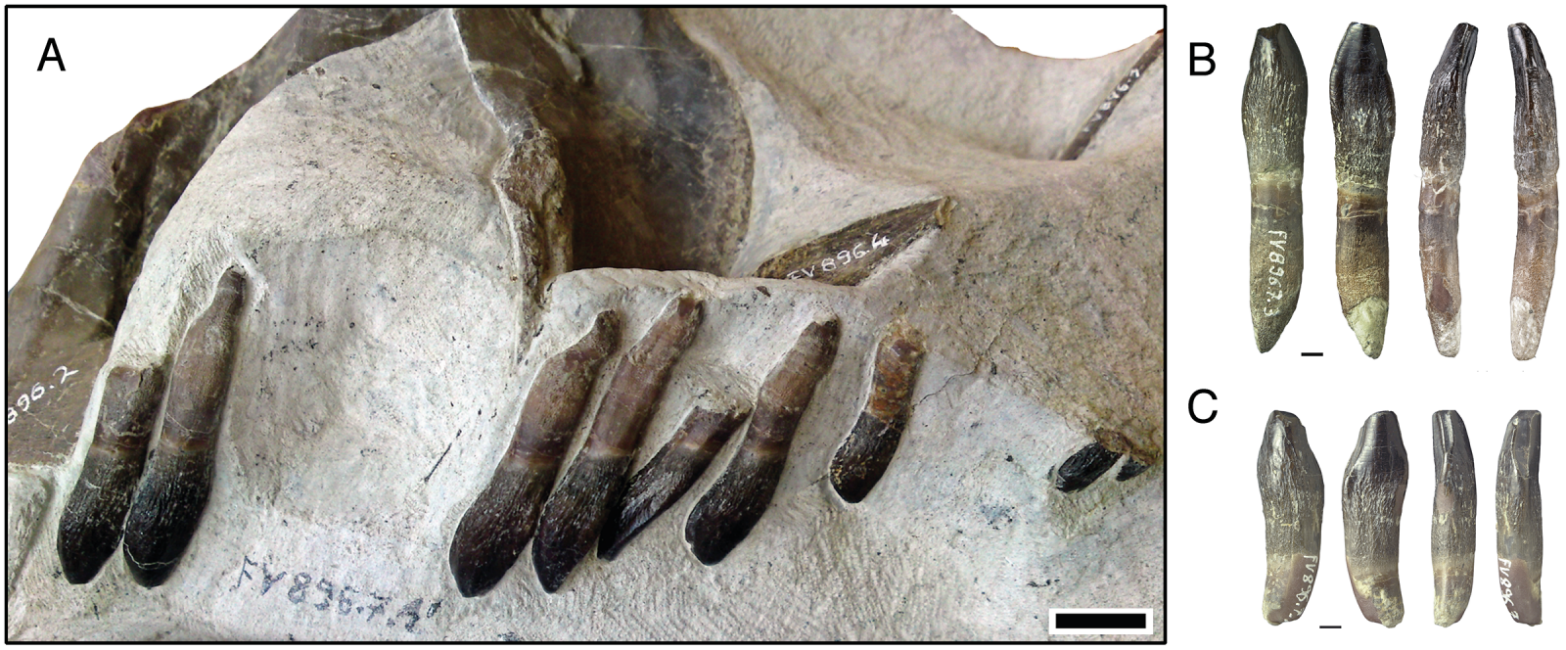
Isolated lower tooth row DfmMh/FV 896.7 of *Europasaurus holgeri*. The first left and right dentary teeth (FV 896.7.2 and FV 896.7.3, respectively) were removed from the specimen during preparation. (A) FV 896.7.1, the part of this ITR remaining in the matrix in labial view. Scale = 10 mm. Note the decrease in size and increase in asymmetry from the mesial to the distal tooth positions. The mesial carina of the teeth extends further basally than the distal carina, indicating that the symphysis of the ITR is located between the two isolated teeth. (B) First left dentary tooth FV 896.7.2 extracted from the ITR in labial, lingual, mesial, and distal view (left to right; scale = 3 mm). (C) First right dentary tooth FV 896.7.3 extracted from the ITR in labial, lingual, mesial, and distal view (left to right); scale = 3 mm.

Based on features such as the degree of wear, root length, tooth exposure, tooth length, tooth size, and the relative position in the alveoli, we assigned developmental stages to the dentition of *Europasaurus* using the approach of Chatterjee & Zheng (2005). F1 to F5 are stages of wear of functional teeth, and R0 to R3 are stages of development of the replacement teeth (Table 1).

**Table 1:** Stages of development (R0 to R3) and wear (F1 to F5) recognized in *Europasaurus* teeth.

Development and wear stages	Description
R0	Small incipient tooth, still sits deep in the jaw bone and is not or hardly visible.
R1	Small incipient tooth that is visible only through the foramen between interdental plates.
R2	Replacement tooth that has advanced into the alveolus.
R3	Replacement tooth that has reached the border of the jaw bone.
F1	Fully developed tooth with intact crown, which shows no signs of wear. The root is still completely intact.
F2	Functional tooth, one of the three wear facets is formed (usually the terminal wear facet). Root resorption is initiated.
F3	Functional tooth. Two of the three wear facets have formed, and between 25% and 50% of the total root length have been resorbed.
F4	Functional tooth. Three wear facets have formed (terminal, main, and side wear facets), and between 50% and 75% of the total root length have been resorbed.
F5	Functional tooth, very advanced wear (terminal, main and side wear facets form a single facet). At least 75% of the root have been resorbed.

The developmental and wear stages of the teeth were plotted on charts and used in determining Z-spacing (Edmund, 1969; DeMar, 1972; Osborn, 1970; Whitlock & Richman, 2013; Schwarz et al., 2015). In Z-spacing, the number of tooth positions between two teeth in the same stage of development is measured. Since all preserved dentaries contain only replacement teeth, and the functional dentary dentition is only represented by a partial tooth row in skull SNHM-2207-R and an incomplete ITR DfmMh/FV 896.7, the Z-spacing diagrams for the dentary cannot be regarded as error-free and only indicate a general trend.

## Results

### Tooth formula

Based on the number of alveoli in the jaw bones (Table 2), the tooth formula for *Europasaurus* is 4 pm + 12 m/13-14 d, as described by Marpmann et al. (2015). All premaxillae clearly show four alveoli, and the only preseved maxilla 12 alveoli. Of the complete dentaries, three (DfmMh/FV 033, DfmMh/FV 034, DfmMh/FV 653) show 13 alveoli. The other complete dentary (DfmMh/FV 291.11), which forms part of the holotype, is slightly larger than the others and shows 14 alveoli. The variability in dentary alveolar counts has been described for other sauropods (Stein et al., 2010), and could represent intraspecific variation. Moore et al. (2018) had suggested that there might be an ontogenetic reduction in the tooth count in sauropods, but there is no evidence for this in *Europasaurus* despite the nearly three-fold size increase represented by the dentaries (see also Marpmann et al., 2015).

**Table 2:** Overview of the teeth preserved in jaw bones.

DfmMh/FV	Left/right	Preservation	Number of alveoli	Preserved teeth	Positions of alveoli that contain teeth	Interdental plates
Premaxillae and maxilla						
032	R	Complete	4	5	1–4 (1)	Yes
061	L	Complete	4	1	2	Yes
291.18	L	Incomplete	2	2	3, 4	–
652.2	L	Complete	4	6	1–4 (1, 3)	Yes
982	R	Complete	4	0	–	Yes
291.17	R	Partially reconstructed	12	9	1–3, 7, 8, 10–12 (8)	Yes
Dentaries						
033	L	Complete	1–13	11	1–8, 10, 11	Yes, 1–11
034	R	Complete	1–13	10	1, 3, 4, 6–10, 12, 13	Yes, 1–10
059	R	Incomplete	4–13	6	4–7, 9, 10	–
092	L	Incomplete	1–7	6	1, 3, 5–7, 9, 10	–
093	L	Incomplete	1, 5–12	4	1, 5, 8, 11	–
094	R	Incomplete	1–8	8	1–8	–
290	L	Partially reconstructed	1–3, 7–13	6	2, 3, 7–9, 11	Yes, 1–3
291.11	L	Complete	1–14	11	1–8, 10, 11, 13	Yes, 1–7
501	R	Incomplete	1–4	3	1, 2, 4	–
653	R	Complete	1–13	15	1–10, 12, 13 (2, 5, 8)	Yes, 1–8
654	L	Incomplete	1–11	9	1–7, 9, 10	–
834.7	R	Incomplete	1–13	11	1–10 (1)	–
1,058.14	L	Partially reconstructed	1–7	1	7	Yes

**Note:**

The numbers in parentheses denote a second replacement tooth in this position.

### Replacement dentition within the jaw bones

#### Premaxillary replacement teeth

**Figure 2:**
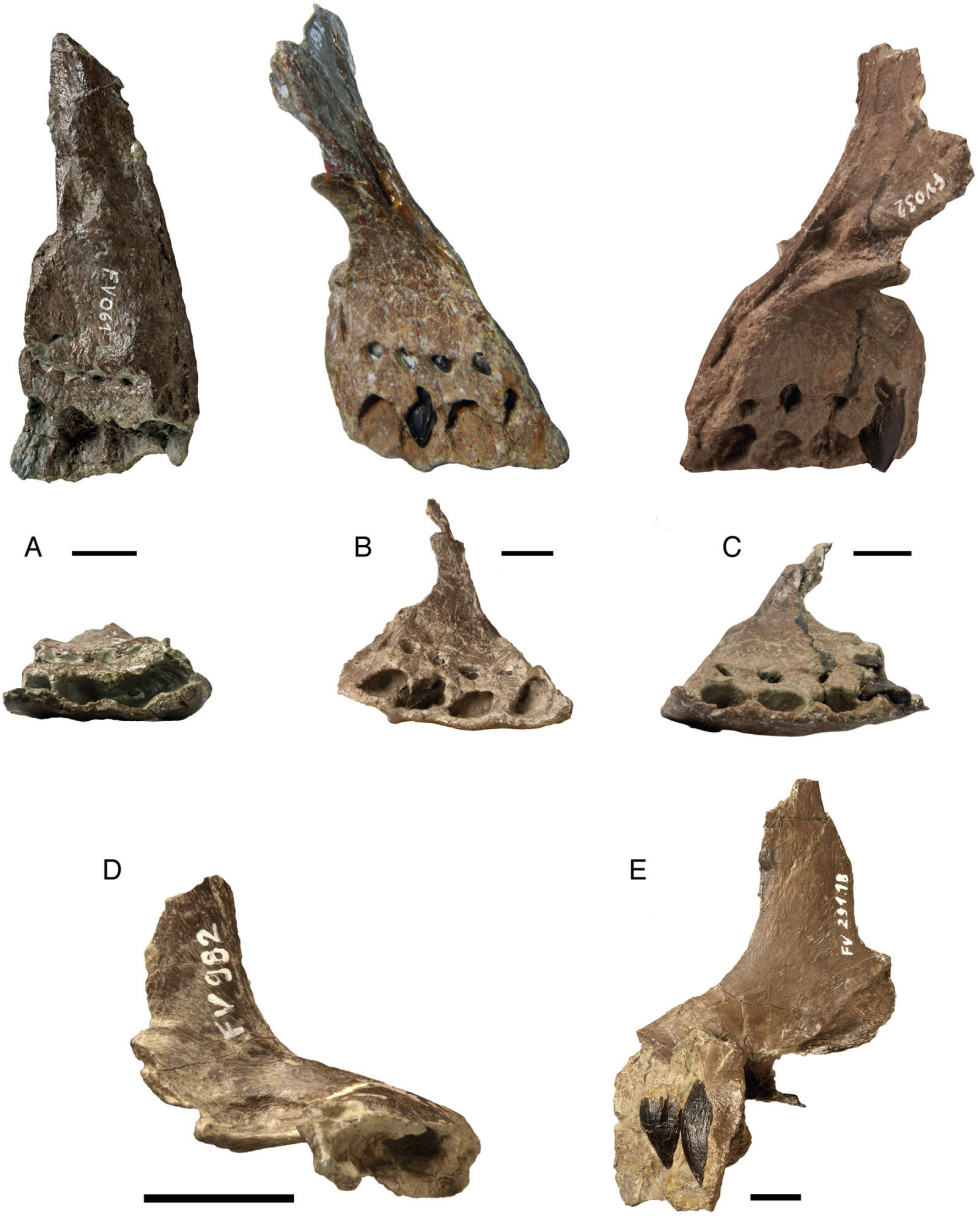
Isolated premaxillae of *Europasaurus holgeri*. All premaxillae show complete alveolar sections and fused interdental plates. (A) FV 061 in lingual and ventral view. (B) FV 652.3 in lingual and ventral view. (C) FV 032 in lingual and ventral view. (D) FV 982 in ventral view. FV 982 is the smallest of the premaxillae and comes from a juvenile individual. (E) FV 291.18 in labial view. Note the replacement teeth at different stages of development in the alveoli of premaxillae FV 032, FV 291.18, and FV 652.3. Scale = 10 mm.

Four of the five premaxillary bones (DfmMh/FV 032, DfmMh/FV 061, DfmMh/FV 652.3, and DfmMh/FV 982) preserve the entire alveolar section (Fig. 2). The premaxilla DfmMh/FV 982 (Fig. 2D) represents the earliest ontogenetic stage (Table 2). The lingual margin of the alveoli is formed by interdental plates, which are completely fused. The replacement teeth are seen in the windows between the fused interdental plates, and it appears as if the teeth form in crypts separate from the alveoli of the functional teeth.

No functional teeth are contained in the premaxillae. The replacement teeth are triangular and slightly asymmetrical in shape, wherein the mesial carina is C-shaped and curved slightly lingually, whereas the distal carina extends almost straight. The labial side is markedly convex, and the tip is strongly curved toward the lingual side. The lingual side of the teeth is slightly concave, and the teeth have a sharp, blade-like appearance. The enamel shows no peculiarities; it is black and has the typical fine wrinkling resembling wrinkling morphotype III of Holwerda, Pol & Rauhut (2015). The wrinkling appears in the higher developmental stages of replacement teeth (Fig. 2).

#### Maxillary replacement teeth

**Figure 3:**
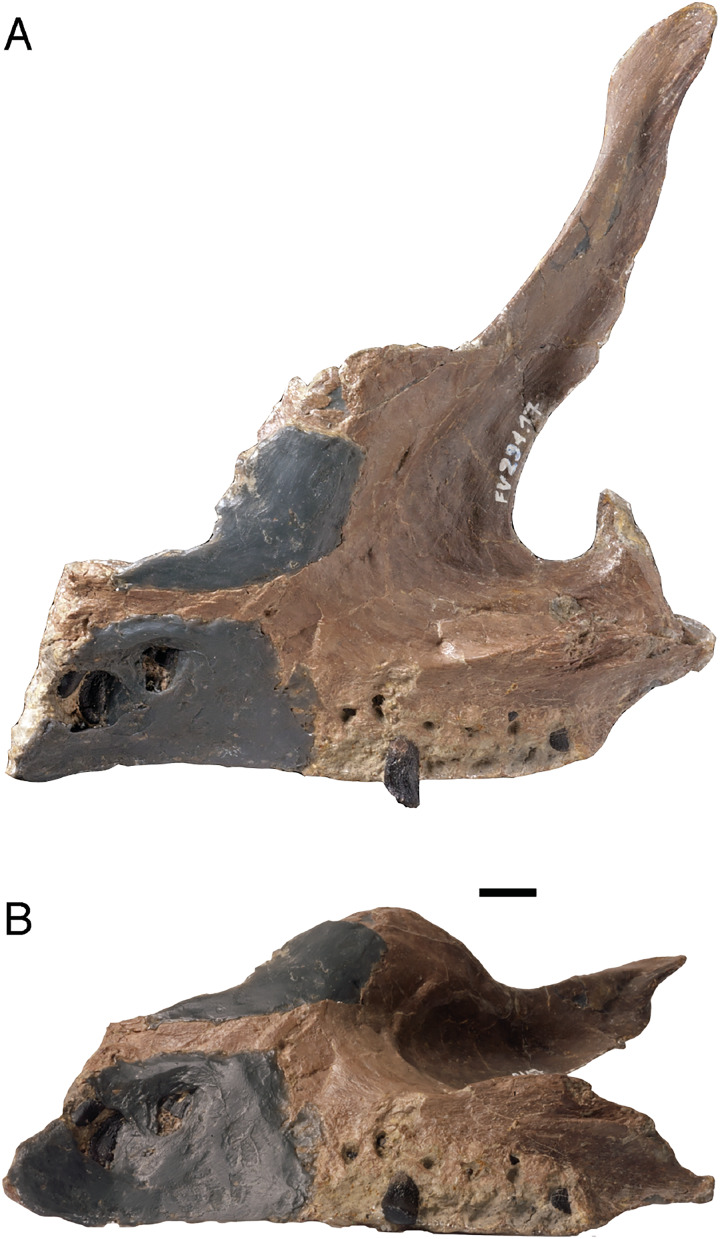
Only known isolated maxilla FV 291.17, part of the holotype of *Europasaurus holgeri*. The first three and last six alveoli with some replacement teeth in place are original, whereas the gray area was reconstructed. It must have contained three to four alveoli. (A) Lingual view, note the fused interdental plate. (B) Ventral view. Scale = 10 mm.

Only one almost complete maxilla of *Europasaurus* is known (DfmMh/FV 291.17), which is part of the holotype (Sander et al., 2006; Marpmann et al., 2015) (Table 2). This maxilla lacks any functional teeth. The alveoli are restricted lingually, and the interdental plates are completely fused. The first three and last six alveoli of the maxilla are clearly seen on the lingual side (Fig. 3), although preservation is not very good. The dimensions of the maxilla and the average distance between the two alveoli indicate that three to four alveoli must have been present in the gap between the two well-preserved parts of the maxilla. We assume a count of three missing alveoli but cannot exclude the possibility that there were four.

Assuming that there were 12 maxillary teeth, replacement teeth are found in almost all preserved alveoli, *i.e*., in positions 1–3, 7, 8, and 10–12, with alveoli 4–6 not preserved (Table 2). The size of the alveoli and the replacement teeth steadily decrease from the mesial to the distal tooth positions. The labial side of the replacement teeth is convex. Lingually, the mesial teeth have a convex surface, whereas the distal teeth have a slightly concave surface. The enamel of the replacement teeth is smooth, and the enamel caps are very pronounced and round, whereas the carinae are not well developed at this early tooth developmental state.

The eighth alveolus contains the most developed maxillary replacement tooth with black enamel and a fine wrinkling pattern (Fig. 3A, Table 2). It is almost completely erupted and has, in contrast to other mesial teeth, an asymmetrical shape. In lingual view, the crown tip is positioned far distally so that the overall shape of the tooth crown appears more rectangular than triangular. The replacement tooth found in the last alveolus shows an even more pronounced asymmetry. Generally, the asymmetry increases from the mesial to the distal tooth positions, also seen in the dentary tooth row. Maxillary teeth differ from the dentary teeth in that the long axis of the tooth is curved lingually, producing a C-shape.

#### Dentary replacement teeth

**Figure 4:**
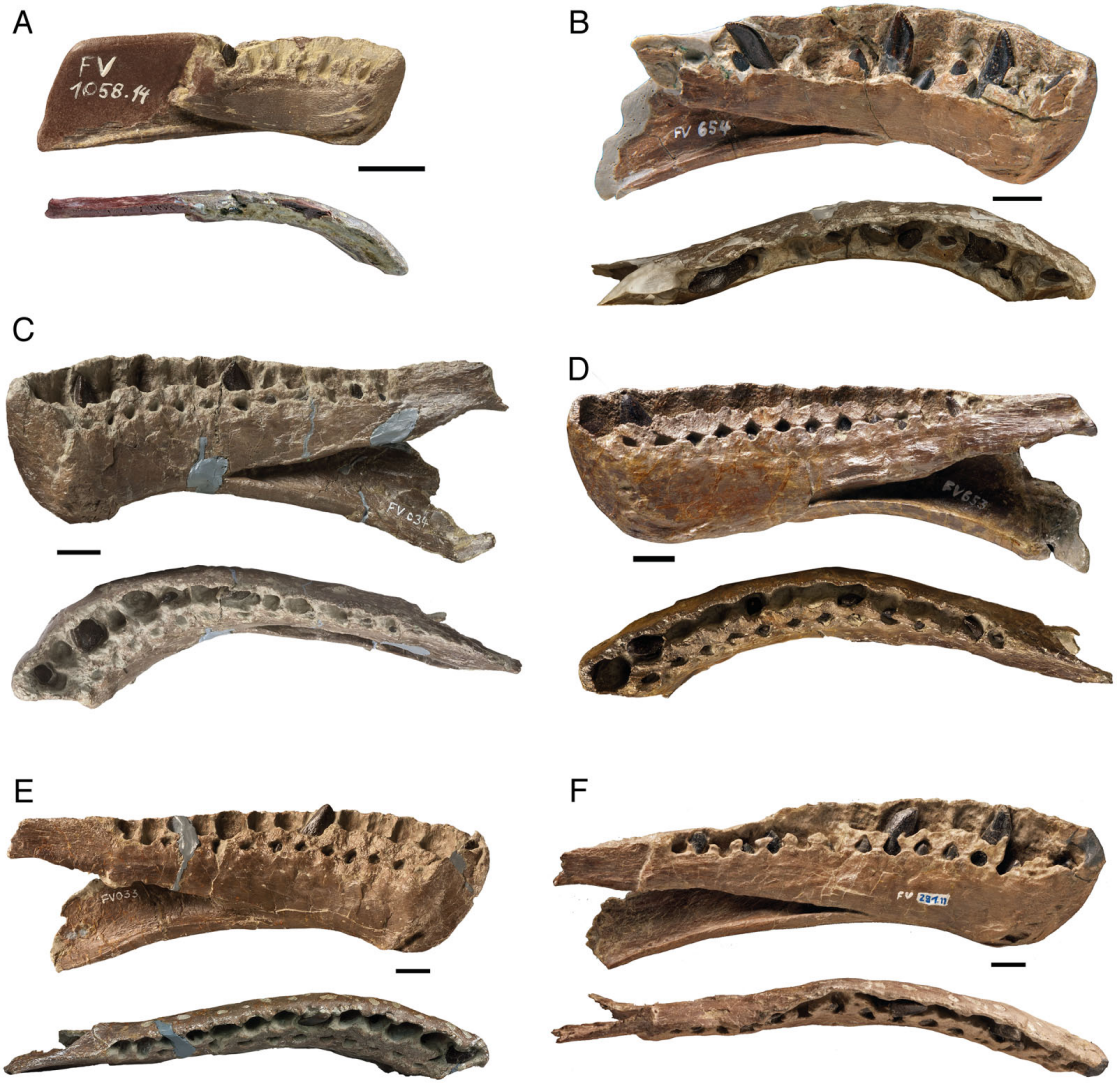
A growth series of dentaries of *Europasaurus holgeri* in lingual and dorsal view. Functional teeth were lost before burial, revealing varying numbers of replacement teeth at different stages of development. (A) FV 1058.14, the smallest and ontogenetically earliest dentary. (B) FV 654. (C) FV 034. (D) FV 653. (E) FV 033. (F) Largest dentary FV 291.11 with 14 alveoli, one more than the other complete dentaries (see Table 2). The fifth tooth of FV 291.11 is rotated by 180° in its alveolus, possibly representing a pathology. Scale = 10 mm.

Of the 13 dentaries of different ontogenetic stages, four are complete, and nine are incompletely preserved (Fig. 4, Table 2). The dentaries represent an approximately three-fold size increase from smallest to largest (Fig. 4). No functional teeth are contained in the dentaries. Almost all replacement teeth are in the bell stage without a deeply and firmly anchored root, but bear an enamel cap. The enamel is dark, almost black, and lacks wrinkling. Only the most advanced replacement teeth (R3) that protrude beyond the dorsal margin of the dentary have a slight wrinkling of the enamel, and the root can be identified.

The crown of the replacement teeth is triangular in shape in labiolingual view and slightly asymmetrical. The mesial carina extends further basally than the distal carina and forms a distinct curve, while the distal carina is straight. The visible part of the labial surface is strongly convex, whereas the lingual surface has weak, elongated concavities at the carinae, which are separated by a convex bulge occurring in the middle of the lingual surface of the tooth crown.

In very immature teeth (tooth crown only), which are even more deeply seated in the jaw, this lingual concavity hardly appears, or occurs only on the mesial carina. In further developed teeth, both the mesial and the distal carina are bordered by the longitudinal groove. The tip of the teeth is slightly inclined lingually. The replacement teeth in the distal alveoli differ in shape from the replacement teeth of the more mesial alveoli. Both the mesial and distal carina are quite extensive; the tip is slightly flattened and slightly inclined lingually. The distal teeth show increasing asymmetry (Fig. 4). The carinae and the tips of immature replacement teeth are smooth and rounded compared to those of later developmental stages.

The size of replacement teeth in the dentaries and the diameter of the alveoli decrease steadily from the mesial to the distal tooth positions. A striking feature of the dentaries is that the front replacement teeth (alveoli 1–4) have a strong rotation about their long axis (apicobasal axis) of about 45° from the lateral margin of the jaw, with the mesial carina rotated lingually and the distal carina rotated labially. This long axis rotation decreases posteriorly. The replacement teeth in alveoli 5–8 are only rotated by 15°, whereas the replacement teeth of the rear positions are positioned in parallel to the long axis of the jaw, with the mesial carinae oriented fully mesially and the distal carinae oriented fully distally. The teeth of the dentaries also show a distinctive ‘en echelon’ pattern, *i.e*., closely spaced teeth in which the distal carina labially overlaps the mesial carina of the succeeding tooth (Wilson, 2002) (Fig. 4).

Almost all replacement teeth in the dentaries show clear and very well formed denticles, mostly only on the mesial carina, but also partly on both carinae. Only well-developed teeth in stage R3, which have already developed wrinkling on the enamel, show no or only very indistinct denticles on the mesial carina. This suggests that denticles are lost as enamel thickness increases during tooth growth.

Very immature replacement teeth located mesially show mostly coarse denticles, five to six in number, whereas older replacement teeth have only three to four poorly defined denticles on the mesial and distal sides. In dentary DfmMh/FV 291.11, the mesial replacement teeth have no denticles. In contrast, both mesial and distal sides of the distal replacement teeth have denticles (Fig. 4A). In dentary DfmMh/FV 654, the replacement teeth in positions 3, 6, and 9 are of approximately the same stage of development and can thus be easily compared. The tooth in the third position shows only very indistinct denticles on the mesial carina, whereas the tooth in the sixth position has five distinct denticles. For the ninth tooth, six denticles are present both on the mesial and distal sides.

A pathology occurs on the fifth replacement tooth in the dentary DfmMh/FV 291.11 (Fig. 4A). This tooth is rotated by 180° in the alveolus, its convex side facing lingually.

### Dentition of partially articulated skull SNHM-2207-R

**Figure 5:**
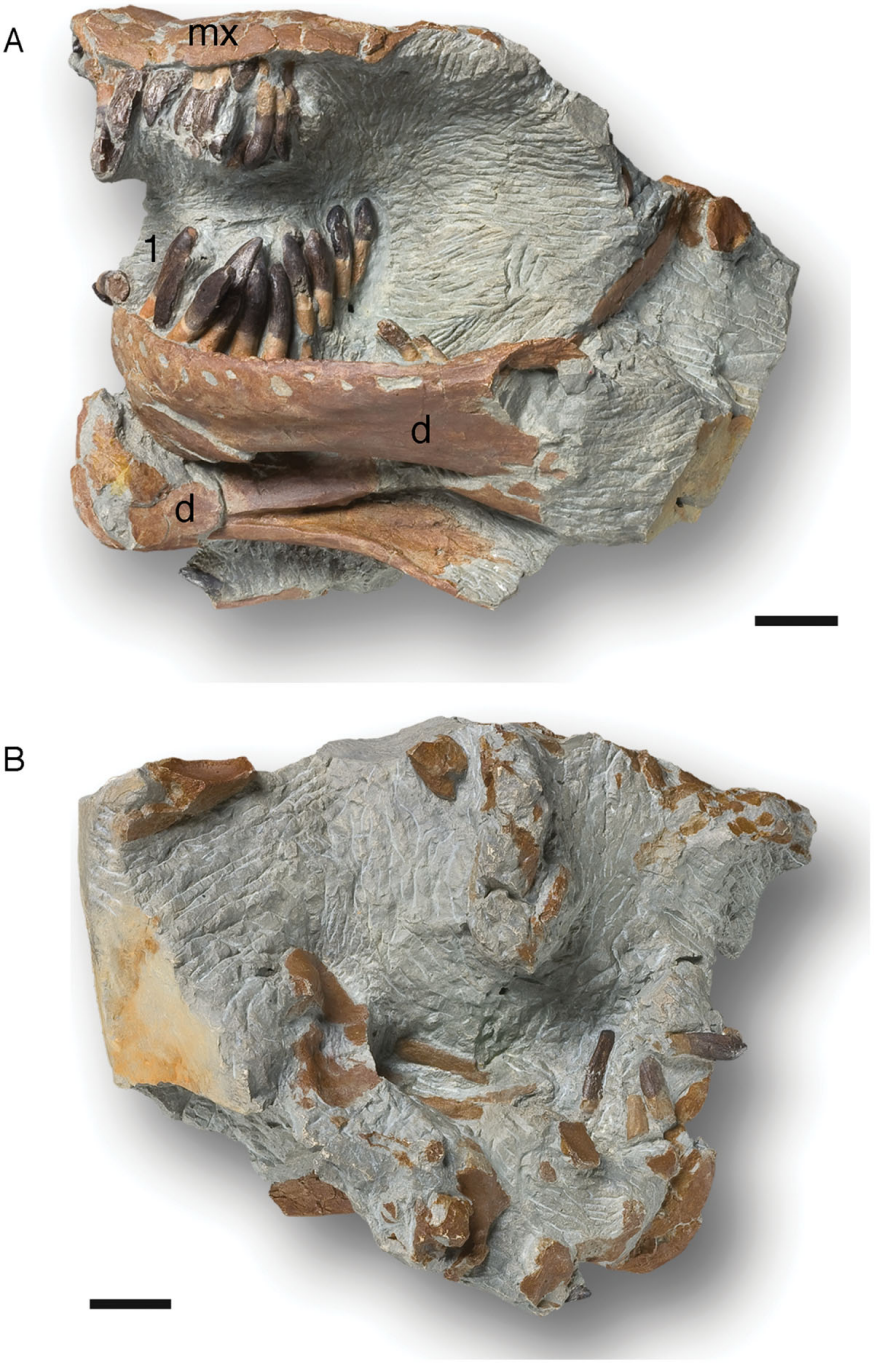
Cast (NLMH 105.996) of the partially articulated skull SNHM-2207-R of *Europasaurus holgeri*. Both dentaries and the posterior part of the left maxilla are preserved. The maxillary teeth are all heavily damaged, but the last two teeth are well preserved in the matrix next to the maxilla. The 3rd and 6th teeth are replacement teeth, the rest are functional teeth. The left dentary possesses 13 alveoli and 12 functional teeth. The right dentary has not been completely freed from the sediment, but six associated teeth have been uncovered. (A) Left side in lateral view. (B) Right side in lateral view. Abbreviations: d, dentary; mx, maxilla; 1, first tooth of the left dentary. Scale = 20 mm.

Both the original (SNHM-2207-R) and a cast of an earlier preparation stage (NLMH 105 996) of this partially articulated skull were studied (Fig. 5). In addition to various skull bones (Marpmann et al., 2015), both dentaries and the posterior part of the left maxilla are preserved.

The fragmentary left maxilla has a preserved length of 54.1 mm. All the maxillary teeth are present, yet most are not well preserved, and the crowns are only fragmentary. Although well preserved, the last two teeth are not in their alveoli, but are embedded in sediment nearby. They show a marked crown asymmetry in labiolingual view which is best described as S-shaped. The base of the crown is set at an angle to the root, and the tip of the crown similarly is angled against the more basal part, but in the opposite direction (Fig. 5). Teeth 3 and 6 are replacement teeth of stage R3. The remaining teeth are functional teeth that protrude further from the jaw. As with the teeth in the dentary, the tooth necks are exposed, and the transition from root to crown is about 5 mm above the lateral mandibular margin (Fig. 5).

The left dentary is 124.5 mm long and 40.1 mm high in the region of the third alveolus. Thirteen alveoli can be recognized, but only 12 clearly identifiable teeth are present. The roots of the teeth are circular to oval in cross section. Apical from the crown base, the cross section of the tooth is lens-shaped. The crown expands mesiodistally, whereas the mesial carina always extends further basally than the distal carina. The lingual side is flattened, whereas the labial side is convex. Every tooth has a slightly asymmetrical appearance, which increases from the mesial to the distal tooth positions (Fig. 5). A longitudinal bulge or ridge on the labial side is mesially shifted in the direction of the carina, whereas the top of the crown and the crown base are moved distally.

Independent of stage, the size of the teeth in the left dentary decrease steadily from mesial to the distal. The first four dentary teeth are still preserved in the alveoli, but are strongly shifted posterolingually. The second tooth is very immature, only the crown being visible (stage R3), and it has not reached the occlusal line of the tooth row yet. The other three teeth, located in the alveoli, protrude much further from the jaw. The transition between the crown and root in these three teeth is about 6 mm above the labial mandibular margin, and the neck of the teeth is exposed.

The first tooth in the left dentary shows a slightly inclined terminal wear facet on the crown tip towards the labial side. Toward the distal carina, the main wear facet is advanced, though partially covered in sediment (stage F3). The situation is similar in the crown of the third tooth. It also shows an almost horizontally directed terminal wear facet, a labial patch of polished enamel, and a significant main wear facet. On this tooth, an incipient side wear facet on the mesial carina is also observed. This tooth shows stage F3–4. Teeth 5–10 are displaced from the alveoli but retain their proper arrangement in the sediment, thus forming a partial ITR. The last two teeth, number 12 and 13, are still firmly anchored in the alveoli, although they were reoriented obliquely and anteriorly by taphonomic processes. Their tooth crowns are damaged.

The pattern of tooth replacement is from the distal to the mesial tooth positions, starting at the 10th alveolus. The 5th, 8th, and 11th alveoli contain replacement teeth in stage R2. The tips of these teeth are already far advanced into the alveolus, but have not yet reached the margin of the jaw. The teeth show stages F3–4, and the roots of the corresponding functional teeth 5 and 8 show advanced resorption, being on the verge of replacement by new teeth. The functional tooth from alveolus 11 is missing.

The right dentary is not completely freed from the sediment, and bone fragments cover it. It is 122.6 mm long, and six associated teeth are located nearby. Five of them, which are completely detached from the alveoli, consist only of fragments. The single tooth remaining in the alveoli is a replacement tooth of stage R3.

### Isolated upper tooth row DfmMh/FV 580.1

**Figure 6:**
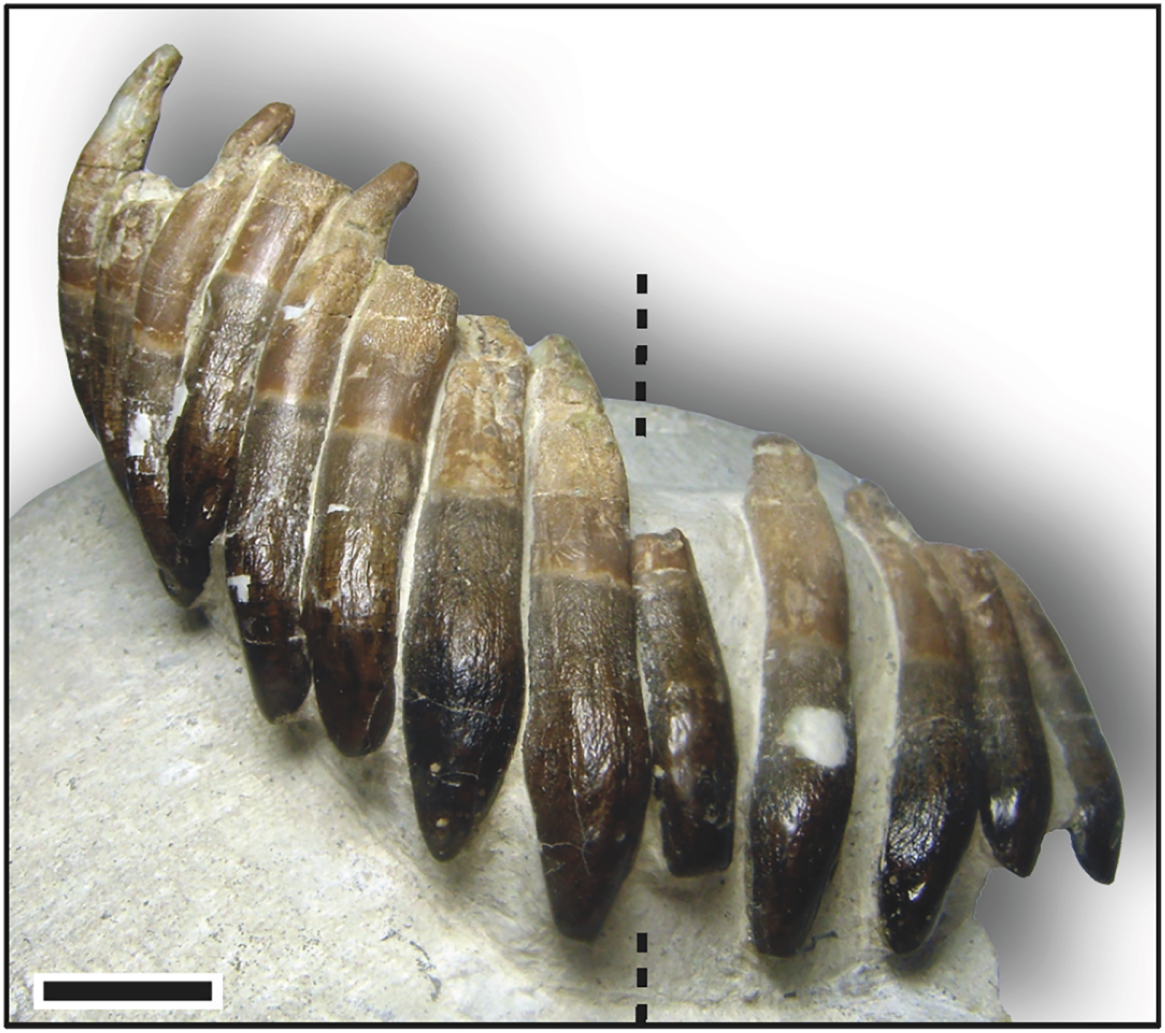
Isolated upper tooth row DfmMh/FV 580.1 of *Europasaurus holgeri* in labial view. The dashed line indicates the location of the symphysis. Based on the comparison with the jaw bones, this tooth row includes the first left maxillary tooth, the four left and right premaxillary teeth, and the first four right maxillary teeth. Note that in the first left premaxillary tooth, the root was resorbed so much that it could not have anchored the tooth in the jaw. The tooth must have only been kept in position by connective tissue. Scale = 10 mm.

ITR DfmMh/FV 580.1 contains 13 functional teeth and covers a total area of about 100 × 60 mm (Table 3). Comparison with the partially articulated skull indicates that the ITR must belong to an upper jaw (Fig. 6). The longitudinal axes of all the teeth in this ITR are more or less parallel to each other, so it can be assumed that the teeth are preserved in the original position. The teeth show the same morphology as the teeth of the partially articulated skull, and the location of the symphysis in the ITR runs between the eighth and ninth preserved tooth, counted from the left along the ITR in labial view (Fig. 6). This means that the tooth row includes the first left maxillary tooth, the four left and right premaxillary teeth, and the first four right maxillary teeth.

**Table 3:** Measurements of the two isolated tooth rows.

Tooth	Total length	Crown length	Crown width	Distance	Wear stage
DfmMh/FV 580.1					
1	36.6	19.3	n.m	n.d	F2
2	25.6	20.3	n.m	n.d	F4
3	40.0	20.7	n.m	n.d	F2
4	30.0	20.5	n.m	n.d	F4
5	44.5	23.0	n.m	n.d	F2
6	37.2	22.7	n.m	n.d	F4
7	45.6	25.1	n.m	n.d	F1
8	48.2	24.4	8.5	n.d	F2
9	25.4	22.0	n.m	n.d	F5
10	46.9	24.2	8.8	n.d	F2
11	45.8	24.1	8.1	n.d	F1
12	30.3	20.6	7.3	n.d	F4
13	34.4	20.1	7.5	n.d	F4
DfmMh/FV 896.7					
1	33.0	n.d	8.3	n.d	F4
2	44.2	n.d	7.7	7.9	F2
3	29.6	n.d	n.m	n.d	F4
4	45.0	n.d	n.m	n.d	F3
5	40.1	n.d	8.9	n.d	F2
6	40.5	n.d	n.m	7.3	F1
7	26.3	n.d	n.m	8.9	F4
8	34.0	n.d	7.5	8.7	F1
9	22.3	n.d	n.m	11.9	F1
10	7.8	n.d	n.m	n.d	F4
11	4.2	n.d	n.m	3.6	F2

**Note:**

The distance between two teeth is always measured from the mesiodistal center of a tooth to the mesiodistal center of the last tooth. All measurements in mm. n.m., not measurable; n.d., not determined.

In DfmMh/FV 580.1, each individual tooth is rotated by 20–30° from the tooth row axis, creating an ‘en echelon’ pattern. The mesial carina of the teeth points lingually, and the distal carina points labially, indicating that the mesial side of the following tooth labially overlaps the distal side of the preceding tooth (Fig. 6).

The teeth are exposed from their convex labial side, while the lingual side and sometimes the carinae of the teeth are still covered by sediment. The longitudinal axis of the teeth is strongly curved labiolingually, approximately forming a C shape, because the apex of the crown and the tip of the root point lingually. The roots of the teeth in this ITR are resorbed to varying degrees, and the tips and carinae of the teeth are considerably abraded.

Some of the teeth of this tooth row show a large depression in the enamel, a few millimeters in size, on the labial side in the upper part of the crown. These depressions are pronounced, especially in the first right premaxillary tooth, first and second right maxillary teeth, first and second left premaxillary teeth, and first left maxillary tooth.

In addition, the roots of the teeth show a peculiarity; in labial view, the longitudinal axis of the root runs vertically from the base of the crown to the root tip, but in the last section of the root, the longitudinal axis forms a slight S-curve with the mesially pointed root tip (Fig. 6). This S-shaped configuration of the root tip in labiolingual view is particularly pronounced in the fourth, right premaxillary tooth and the first and third, right maxillary teeth. The crown length and crown width decrease steadily from the mesial to the distal tooth positions, and the S-shaped asymmetry in labiolingual view increases.

#### Right premaxillary teeth of DfmMh/FV 580.1

The first right premaxillary tooth is almost completely preserved. The root is complete and shows slight resorption traces only on the labial side of the root tip. The crown is worn and slightly lingually flattened. The terminal wear facet has not spread onto the mesial and distal carinae, and the tooth can thus be assigned to stage F2. The second right premaxillary tooth seems to be very immature. The root shows no resorption traces nor does the top of the crown show any trace of wear. The tooth must have advanced into the functional position shortly before death and is assigned to stage F1. The third right premaxillary tooth is heavily worn. About half of the root is resorbed, and the tooth crown shows distinct wear traces (Fig. 6). The terminal wear facet is flat, inclined lingually, and extends to the distal carina with an extensive side wear facet. The tooth is in an advanced stage F4. The root of the fourth right premaxillary tooth appears completely intact, and only slight resorption at the root has begun on the labial side. The crown has some slight terminal wear, and the enamel appears smooth and polished. Based on these features, especially the stage of development of the wear facets, this tooth is slightly less mature than the first right premaxillary tooth, but due to terminal wear, it is also assigned to stage F2.

#### Right maxillary teeth of DfmMh/FV 580.1

The first right maxillary tooth clearly shows advanced resorption of the root. Nearly two-thirds of the root is resorbed, and the crown shows distinct wear traces (Fig. 6). The terminal wear facet is inclined on the top of the crown, flattened lingually, and gives way to the side wear facet along the distal carina. The mesial carina is covered by sediment. Developmentally, this tooth is slightly older than the third right premaxillary tooth and is also assigned to stage F4.

The second right maxillary tooth shows an intact root without resorption traces. The crown appears to be slightly flattened on top, and there is an incipient lingually inclined terminal wear facet. Labially, the enamel is well polished. Sediment cover of the crown prevents clear observations of surface wear, but the tooth probably is stage F2.

On the third right maxillary tooth, significant resorption of the root is noticeable. It is more advanced than the first right maxillary and the third right premaxillary tooth. The developmental stage of these three teeth thus decreases in sequence from the distal to the mesial tooth positions. This tooth shows a distinct wear surface, which stretches beyond the distal carina (stage F4). The terminal wear facet seems present and slightly tilted lingually, but is covered by sediment. Wear on the side is not as pronounced as with the other two teeth. Although the resorption of the roots increases from the mesial to the distal tooth positions, the wear facets seem to become less pronounced.

The fourth right maxillary tooth is the last tooth located in the right maxillary part of the isolated tooth row. Its root is intact and the crown shows minimal wear traces, only a smoothing of the enamel. It is difficult to see whether the formation of the terminal wear facet had already begun, but it does appear that the crown tip is slightly flattened. This tooth is assigned to stage F2.

Clearly, a replacement pattern can be seen in the teeth of the upper right quadrant of ITR DfmMh/FV 580.1. Every second tooth is associated with a developmental series, so alternating zahnreihen occur on odd and even tooth positions. The teeth become less mature from the distal to the mesial tooth position.

#### Left premaxillary teeth and first left maxillary tooth of DfmMh/FV 580.1

The first left premaxillary tooth is the most worn tooth in this ITR. The root is almost completely resorbed, only a small region below the transition from root to crown remains (Fig. 6). The terminal wear facet is very pronounced and inclined lingually. The side wear facet, which extends far down the crown base, meets the terminal wear facet on the distal carina, and the tooth is therefore assigned to stage F5.

The apex of the second left premaxillary tooth shows beginning resorption on the lingual side. On the crown, the terminal wear facet is flattened and inclined lingually, and polishing of the enamel can be observed (stage F2).

The third left premaxillary tooth seems fully intact. The root shows no resorption traces, and the tooth crown enamel is only slightly polished (stage F1). A striking feature of this tooth is a longitudinal, elongated concave groove along the tooth root, which extends from the root tip over almost the entire root length in the direction of the crown base (Fig. 6). The groove may have resulted from the collapse of the pulp cavity which in a tooth of stage F1 would have been large.

The fourth left premaxillary tooth shows a very pronounced terminal wear facet that extends into the distal side wear facet and mesial main wear facet. The wear surface is extensive, and two-thirds of the root is already resorbed (stage F4).

The first left maxillary tooth is only slightly less mature than the fourth left premaxillary tooth and is also in the F4 stage. Half of the root is resorbed, and all wear facets are quite pronounced.

### Isolated lower tooth row DfmMh/FV 896.7

The ITR DfmMh/FV 896.7 consists of a total of nine associated dentary teeth (Table 3) and two additional, smaller teeth (no number) on a block of sediment. However, the degree of articulation is not as high as in the previously described ITR. The nine teeth are preserved with eight other bones, including several cervical ribs and a cervical vertebra (Fig. 7A). To reveal their morphology in full, the first left dentary tooth (DfmMh/FV 896.7.2, Figs. 1, 7C) and the first right dentary tooth (DfmMh/FV 896.7.3, Fig. 7B) were separated from the block during preparation, but they are considered to belong to this ITR.

This ITR also shows the ‘en echelon’ pattern of the teeth as in ITR DfmMh/FV 580.1 (Figs. 6, 7). As observed in the partially articulated skull SNHM-2207-R and ITR DfmMh/FV 580.1, ITR DfmMh/FV 896.7.1 shows a significant decrease in crown length and width from the mesial to the distal tooth positions, whereas the asymmetry of the teeth increases steadily in distal direction (Fig. 7).

The visible side of the tooth row is strongly curved and thus represents the labial side, whereas the lingual side is hidden in the sediment. The long axis of the teeth is straight, with barely any curvature of the tip to the lingual side of the tooth. These teeth also have an asymmetrical tooth crown, where the mesial carina is extended further than the distal carina, indicating that the symphysis must have been located between the two isolated teeth DfmMh/FV 896.7.2 and DfmMh/FV 896.7.3.

The first to sixth right dentary tooth are most certainly part of the ITR, but the affinity of the two smaller teeth (located about 25 mm away from the rest) is unclear (Fig. 6). The gap between the sixth tooth and the first of the two smaller teeth and the size difference seems to be too large for the two smaller teeth to be consecutive teeth of this ITR.

#### Right dentary teeth of DfmMh/FV 896.7

The first right dentary tooth (Fig. 7A), isolated from the block shows some resorption of the root (Fig. 7B). The crown tip is flattened and shows a terminal wear facet, indicating wear stage F3.

The root of the second right tooth (in place) appears to be completely intact (Fig. 7A). On the crown, the side wear facet has a slight mesial wear surface (stage F2). The distal carina and the crown tip are covered by sediment, preventing further observations.

The third right dentary tooth is a relatively immature tooth with a complete root (Fig. 7A). The crown shows no wear traces, and the labial enamel has no smoothing or polishing (stage F1). Four denticles are present on the mesial carina.

The fourth right tooth has a flat wear surface on the crown. Its root is almost completely resorbed (stage F4). A striking feature of this tooth is that it is vertically misaligned compared to the rest (Fig. 7A). Its mesial carina is rotated strongly lingually, whereas the distal carina is rotated labially. In addition, the tip of the tooth has shifted in the direction of the preceding third tooth, and the crown base has shifted in the direction of the succeeding fifth tooth. Thus, the longitudinal axis of the tooth does not extend parallel to the longitudinal axes of the other teeth, and it is likely that this tooth is no longer in its original position.

The position of the fifth right tooth corresponds to that of the remaining teeth. The tooth is slightly older than the third one and already has a weak polishing of the labial enamel. Nevertheless, on the mesial carina, denticles can be identified, and the root is still intact. Thus it is still assigned to stage F1, but differs from the preceding right teeth in its strong asymmetry, which manifests itself as an S-shaped curve along the tooth long axis in labiolingual view. This asymmetry is also pronounced on the sixth right tooth. The tip of the root is missing due to the sediment block being broken off at this position, and the carinae cannot be observed due to sediment cover.

#### Left dentary teeth of DfmMh/FV 896.7

DfmMh/FV 896.7.2 (Figs. 1, 7C), the first left dentary tooth, has a highly advanced root resorption stage and only about one third of the root is left. At the top of the crown, over the entire width, a prominent, steeply angled and flat terminal wear facet can be seen, which only affects the enamel on the labial side. The terminal wear facet spreads out toward the main and side wear facets in the distal and mesial directions. This tooth can thus be assigned to stage F4.

The second left tooth crown shows a slightly flattened top with a flat terminal wear facet (stage F2). Only one-third of the root of the third and last preserved left dentary tooth is present. The enamel of the crown on the labial side is polished, and the crown tip has a flat, terminal wear facet. On the distal and mesial carina, the main and side wear facets are already quite pronounced, and both facets meet the terminal wear facet without any distinct border, creating one large wear surface (stage F4).

### Isolated teeth

**Figure 8:**
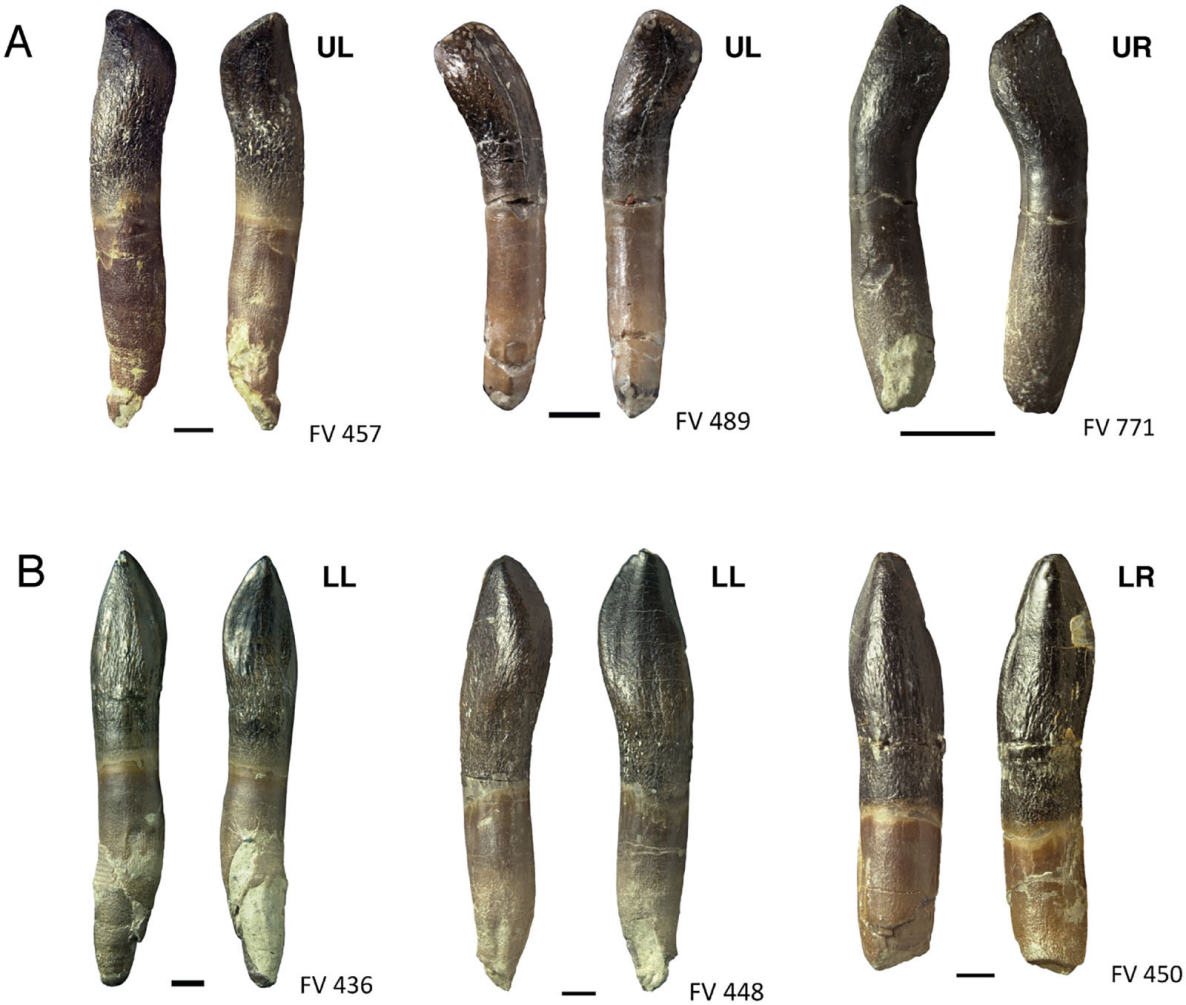
Comparison and asymmetry of upper and lower, mesial and distal teeth of *Europasaurus holgeri*. (A) Isolated distal maxillary teeth FV 457, FV 489, and FV 771 in labial and lingual view. The S-shaped distal displacement of the crown tip is clearly recognizable. (B) Isolated lower mesial teeth FV 436, FV 448, and FV 450 in labial and lingual view. The crown is very symmetrical, and the crown tip is hardly displaced mesiodistally. Scale = 3 mm. Jaw quadrant abbreviations: UR, upper right; UL, upper left; LR, lower right; LL, lower left.

**Figure 9:**
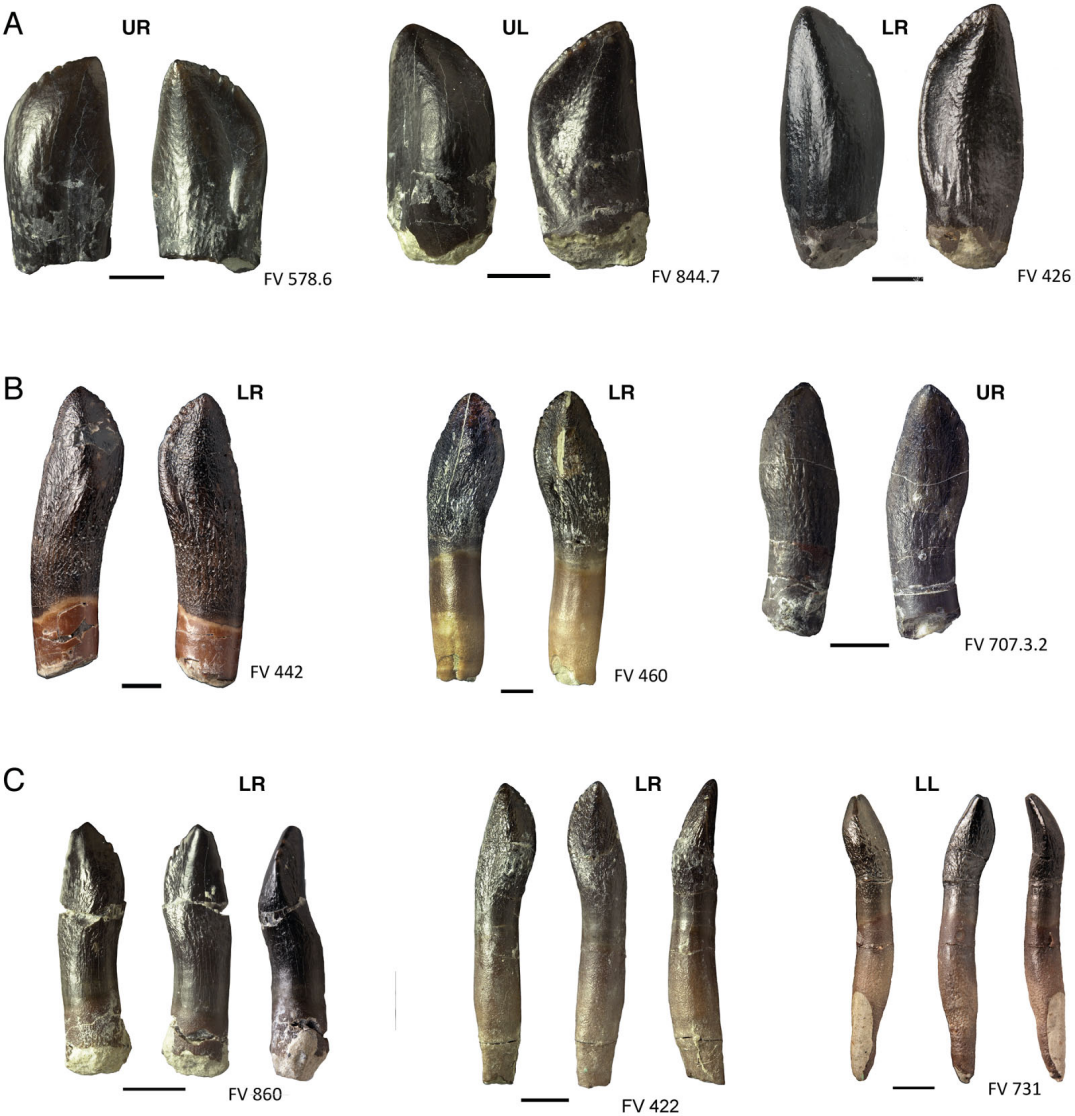
Various forms of denticles on the teeth of *Europasaurus holgeri*. (A) Isolated replacement teeth FV 578.6, FV 844.7, and FV 426, from left to right, in labial and lingual view. The teeth show coarse denticles on the mesial carina. The enamel of the teeth is smooth, the root is not developed. (B) Juvenile functional teeth FV 442, FV 460, and FV 707.3.2, from left to right, in labial and lingual view. They already display wrinkled enamel and indistinct denticles on the mesial carina. The relatively juvenile dentary tooth FP 460 also shows slight denticles at the distal carina. (C) Older, functional teeth FV 860, FV 422, and FV 731, left to right, each in labial, lingual, and mesial view. Their overall appearance is more slender than that of the juvenileer teeth because of root elongation. Tooth FV 860 is the only isolated tooth that shows denticles on the distal carina. The tooth FV 731 is fully developed and shows significant wear instead of denticles. Scale = 3 mm. Jaw quadrant abbreviations: UR, upper right; UL, upper left; LR, lower right; LL, lower left.

**Figure 10:**
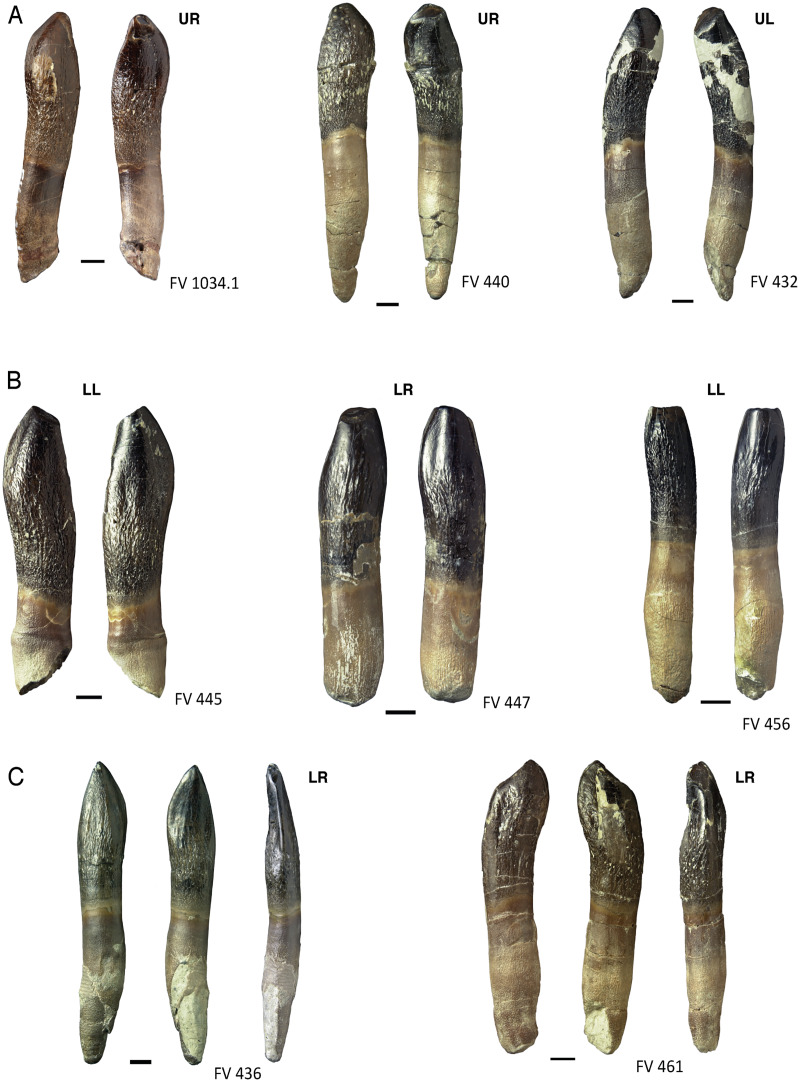
Terminal wear facets on the teeth of *Europasaurus holgeri*. (A) Maxillary teeth FV 1034.1, FV 440, and FV 432, left to right, in labial and lingual view. The terminal wear facets are steeply inclined mesiolingually. (B) Dentary teeth FV 445, FV 447, and FV 456, left to right, in labial and lingual view. The wear facet is horizontal or inclined slightly distolabially. (C) Lower jaw teeth FV 436 and FV 461, left to right, in labial, lingual, and distal view. On both, the wear facets diverge from the common pattern, possibly caused by missalignment of the teeth. Scale = 3 mm. Jaw quadrant abbreviations: UR, upper right; UL, upper left; LR, lower right; LL, lower left.

**Figure 11:**
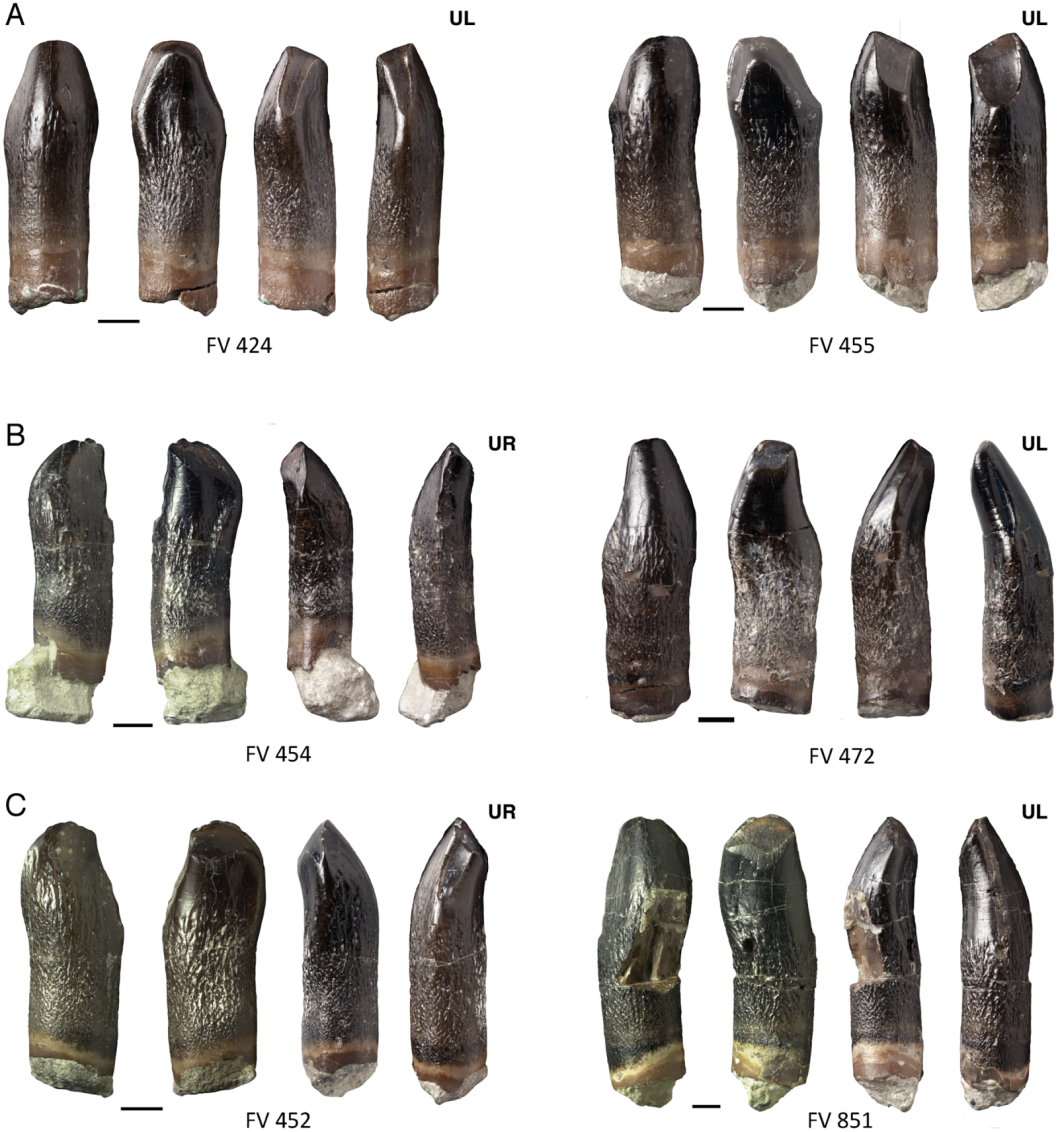
Main and distinct side wear facets of maxillary teeth of *Europasaurus holgeri*. (A) FV 424 and FV 455 in labial, lingual, mesial, and distal view (left to right). FV 424 and FV 455 show terminal, main and side wear facets. (B) FV 454 and FV 472 in labial, lingual, mesial, and distal view (left to right). The side wear facet is not connected to the terminal wear facet. (C) FV 452 and FV 851 in labial, lingual, mesial, and distal view (left to right). The side wear facet exceeds the main wear facet in development and size. Scale = 3 mm. Jaw quadrant abbreviations: UR, upper right; UL, upper left; LR, lower right; LL, lower left.

**Figure 12:**
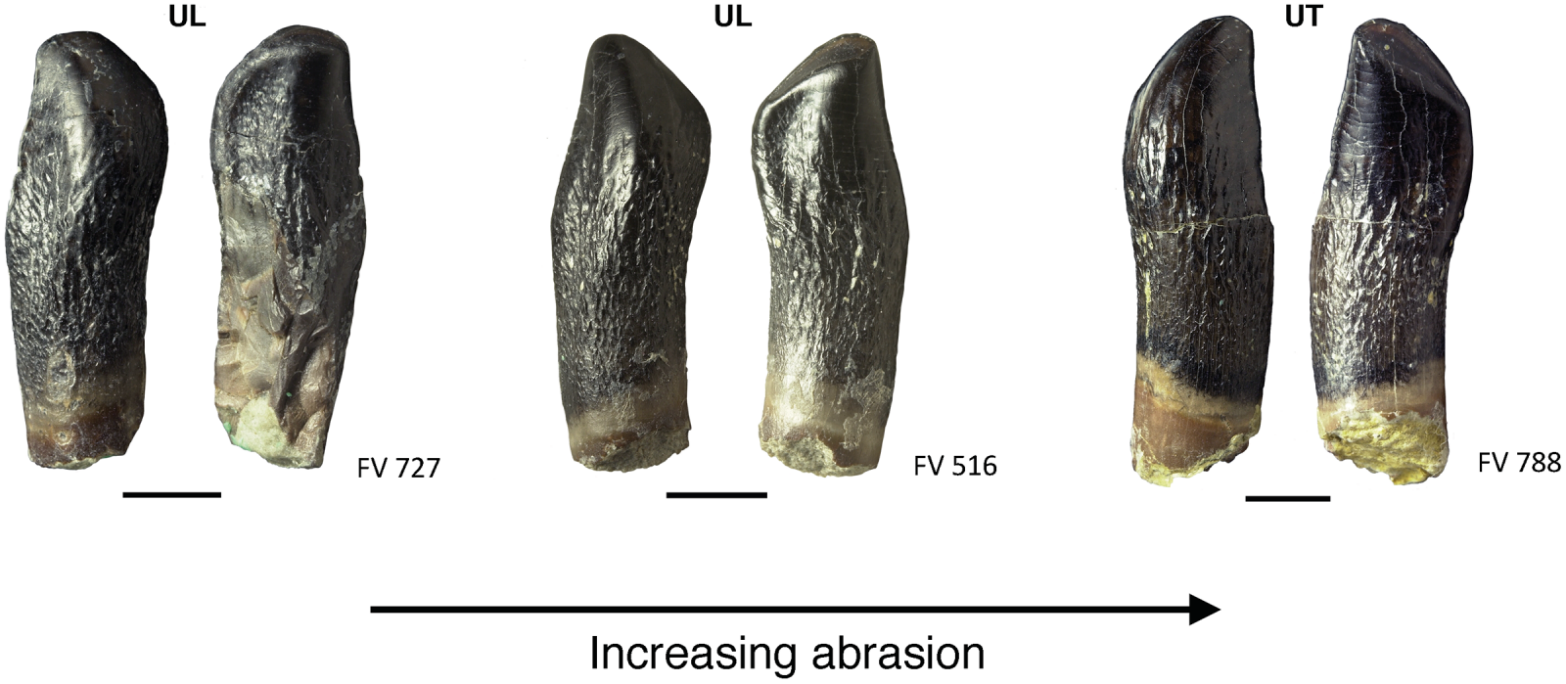
Advanced tooth wear in *Europasaurus holgeri*. Teeth FV 727, FV 516, and FV 788 in labial and lingual view (left to right). The terminal wear facet shifted with increasing wear more and more toward the side of the main wear facet, and together the facets form a teardrop-shaped facet (see also Fig. 10, FV 452 and FV 454). Note the strongly resorbed root in these teeth. Scale = 3 mm. Jaw quadrant abbreviations: UR, upper right; UL, upper left; LR, lower right; LL, lower left.

**Figure 13:**
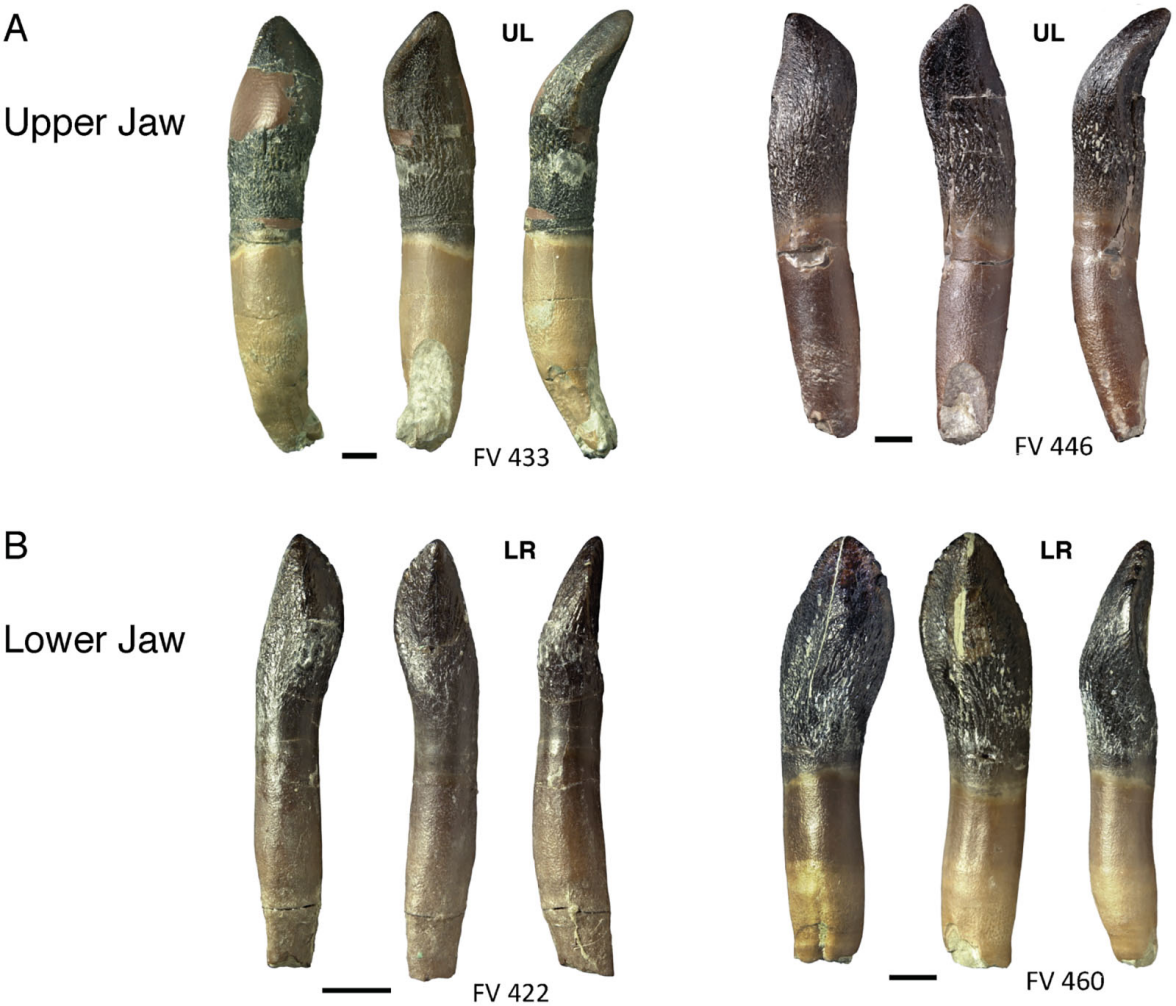
Comparison of upper and lower jaw teeth of *Europasaurus holgeri*. (A) Upper jaw teeth FV 433 and FV 446 in labial, lingual, and mesial view (left to right). Note the C-shaped curvature of the long axis toward lingual. (B) Lower jaw teeth FV 422 and FV 460 in labial, lingual, and mesial view (left to right). The long axis of the tooth is oriented straight and nearly vertically. Scale = 3 mm. Jaw quadrant abbreviations: UR, upper right; UL, upper left; LR, lower right; LL, lower left.

In this section, general individual morphological features and characteristics of isolated teeth are described (Table 4). The isolated teeth (Figs. 1, 8–13) reveal many features, in particular of the lingual side of functional teeth, that cannot be observed fully in the previously described material from skulls and ITRs and hence are described here. A particularly notable morphological feature in 44 teeth is evidence of wear, consisting of enamel polish and tooth wear.

**Table 4:** Measurements, resorption of roots, and wear stages of isolated teeth.

DfmMh/FV	Total length	Crown length	Crown width	SI	Wear stage	TF	MF	SF	Abrasion type	Rooth length	Resorption	Denticles
278	22.4	n.m.	n.m.	n.d.	F?	–	–	–	–	n.m.	n.d.	–
280	21.2	n.m.	n.m.	n.d.	F?	–	–	–	–	8.0	50–75%	–
422	18.5	9.4	3.3	2.85	F1	–	–	–	–	9.1	<25%	4 m
424	19.9	15.6	6.7	2.33	F5	Y	Y	Y	B	4.3	>75%	–
426	17.6	n.m.	n.m.	n.d.	R?	–	–	–	–	n.m.	n.d.	5 m
428	30.3	24.1	8.9	2.71	F4	Y	Y	Y	B	6.2	>75%	–
429	27.4	14.6	6.0	2.43	F1	–	–	–	–	12.8	<25%	3 m
430	34.0	20.8	9.4	2.21	F4	Y	Y	Y	B	13.2	50–75%	–
431	16.5	13.0	4.8	2.71	F2	Y	N	N	A	3.5	n.d.	–
432	40.6	19.9	7.3	2.73	F3	Y	?	N	A	20.7	<25%	–
433	38.9	20.1	7.9	2.54	F1	–	–	–	–	18.8	<25%	–
434	38.7	22.1	8.0	2.76	F3	Y	Y	N	B	16.6	25–50%	–
435	31.4	25.2	9.7	2.60	F1	–	–	–	–	6.2	>75%	–
436	42.0	20.6	7.2	2.86	F2	N	Y	N	B	21.4	<25%	–
437	37.2	n.m.	n.m.	n.d.	F?	–	–	–	–	23.8	<25%	–
438	40.5	22.5	7.8	2.88	F2	Y	N	N	A-B	18.0	n.d.	–
439	39.5	23.0	8.6	2.67	F3	Y	Y	N	A-B	16.5	n.d.	–
440	42.0	20.1	8.3	2.42	F2	Y	N	N	A	21.9	<25%	–
441	44.9	n.m.	n.m.	n.d.	F?	–	–	–	–	20.8	<25%	–
442	26.2	20.8	7.1	2.93	R3	–	–	–	–	5.4	n.d.	5 m
443	28.2	n.m.	n.m.	n.d.	F1	Y	N	N	A	7.7	n.d.	–
444	27.3	18.4	7.2	2.56	F1	–	–	–	–	8.9	n.d.	3 m
445	32.6	22.5	8.2	2.74	F2	Y	N	N	A	10.1	n.d.	–
446	34.5	17.7	6.7	2.64	F1	–	–	–	–	16.8	<25%	3 m
447	30.8	18.6	7.3	2.55	F4	Y	Y	Y	B	12.2	50–75%	–
448	39.0	22.0	7.8	2.82	F2	Y	N	N	?	17.0	<25%	Indistinct
449	27.0	24.5	8.2	2.99	F1	–	–	–	–	2.5	n.d.	–
450	34.0	22.1	7.6	2.91	F2	Y	N	N	?	11.9	n.d.	–
451	32.6	20.4	7.8	2.62	F1	–	–	–	–	12.2	n.d.	–
452	20.8	17.5	7.2	2.43	F4	Y	Y	Y	A-B	3.3	>75%	–
453	22.9	19.7	7.6	2.59	F1	–	–	–	–	3.2	n.d.	3 m
454	17.0	14.8	6.6	2.24	F4	Y	Y	Y	A-B	2.2	>75%	–
455	19.4	17.3	7.3	2.37	F5	Y	Y	Y	B	2.1	>75%	–
456	30.6	14.8	5.5	2.69	F4	Y	Y	Y	B	15.8	<25%	–
457	33.1	16.1	6.1	2.64	F1	–	–	–	–	17.0	<25%	Indistinct
458	29.8	15.9	6.1	2.61	F1	–	–	–	–	13.9	25–50%	Indistinct
459	24.0	21.8	7.6	2.87	F2	Y	N	N	?	2.2	n.d.	–
460	27.5	16.1	6.9	2.33	F1	–	–	–	–	11.4	<25%	4 m
461	33.7	17.4	7.2	2.42	F3	Y	Y	N	A	16.3	25–50%	–
462	38.7	n.m.	n.m.	n.d.	F?	–	–	–	–	18.8	<25%	–
472	23.5	21.2	7.6	2.79	F4	Y	Y	Y	B	2.3	>75%	–
478	22.4	12.8	5.5	2.33	F1	–	–	–	–	9.6	n.d.	4 m
479	25.9	13.9	5.5	2.53	F2	Y	N	N	A	12.0	25–50%	–
486	24.0	12.8	5.1	2.51	F2	Y	N	N	?	11.2	<25%	–
487	24.0	13.8	5.9	2.34	F1	–	–	–	–	10.2	<25%	3 m
488	30.7	12.9	5.7	2.26	F1	–	–	–	–	17.8	<25%	Indistinct
489	23.6	10.7	4.5	2.38	F1	–	–	–	–	12.9	<25%	Indistinct
492.8	14.7	n.m.	n.m.	n.d.	R?	–	–	–	–	n.m.	n.d.	4 m
495.6	15.0	12.2	5.2	2.35	F4	Y	Y	Y	A-B	2.8	>75%	–
496	20.4	11.3	4.9	2.31	F1	–	–	–	–	9.1	25–50%	Indistinct
504	15.5	n.m.	n.m.	n.d.	R?	–	–	–	–	n.m.	n.d.	–
516	12.0	8.5	3.2	2.66	F3	Y	Y	N	A	3.5	n.d.	–
537	16.3	14.9	6.3	2.37	F3	Y	Y	N	A	1.4	n.d.	–
558	42.0	20.8	7.2	2.89	F2	Y	N	N	B	21.2	<25%	–
578.6	10.5	n.m.	n.m.	n.d.	R?	–	–	–	–	n.m.	n.d.	4 m
607	17.7	14.9	5.8	2.57	F1	–	–	–	–	n.m.	n.d.	4 m 3d
636	28.5	18.8	7.5	2.51	F1	–	–	–	–	9.7	<25%	Indistinct
660	20.9	17.1	6.9	2.48	F1	–	–	–	–	3.8	n.d.	Indistinct
662	20.5	18.0	6.8	2.65	F5	Y	Y	Y	B	2.5	>75%	–
663.1	24.6	18.5	7.6	2.43	F3	Y	N	N	A	6.1	50–75%	–
707.3.1	24.8	14.0	4.8	2.92	F2	Y	N	N	?	10.8	25–50%	Indistinct
707.3.2	12.2	9.5	3.5	2.71	F1	–	–	–	–	2.7	n.d.	3 m
726	30.7	n.m.	n.m.	n.d.	F?	–	–	–	–	n.m.	n.d.	–
727	13.6	12.1	4.6	2.63	F2	Y	N	N	A	1.5	n.d.	Indistinct
730	14.4	n.m.	n.m.	n.d.	F5	Y	Y	Y	?	1.8	>75%	–
731	21.5	9.2	3.2	2.88	?	–	–	–	–	12.3	<25%	–
771	12.6	5.8	2.5	2.32	F1	–	–	–	–	6.8	n.d.	3 m
788	19.1	15.2	5.5	2.76	F3	Y	Y	N	A	3.9	n.d.	–
790.6	7.3	7.3	2.9	2.52	R?	–	–	–	–	n.m.	n.d.	4 m
790.7	10.5	9.2	3.2	2.88	F2	Y	N	N	?	1.3	n.d.	3 m
844.7	12.4	n.m.	n.m.	n.d.	R?	–	–	–	–	n.m.	n.d.	3 m
848	23.0	n.m.	n.m.	n.d.	F?	–	–	–	–	n.m.	n.d.	–
851	20.4	17.5	6.5	2.69	F3	Y	N	Y	A	2.9	>75%	–
860	13.6	10.0	3.5	2.86	F2	Y	N	N	?	3.6	50–75%	3 m 2d
865.1	15.6	14.4	5.3	2.72	R?	–	–	–	–	1.2	n.d.	3 m
868.2	14.8	n.m.	n.m.	n.d.	F?	–	–	–	–	n.m.	n.d.	–
869	9.9	n.m.	n.m.	n.d.	F?	–	–	–	–	3.9	n.d.	–
876	9.5	n.m.	n.m.	n.d.	R1-2	–	–	–	–	n.m.	n.d.	3 m
888	11.1	n.m.	n.m.	n.d.	R2	–	–	–	–	n.m.	n.d.	4 m
899	27.7	21.8	7.6	2.87	F1	–	–	–	–	5.9	n.d.	5 m
896.7.2	29.6	21.7	9.0	2.41	F4	Y	Y	Y	B	7.9	50–75%	–
896.7.3	45.0	22.9	8.9	2.57	F2	Y	N	Y	B	22.1	25–50%	–
948	21.1	9.3	3.6	2.58	F2	Y	N	N	?	11.8	<25%	3 m
967	18.7	9.2	3.7	2.49	?	–	–	–	–	9.5	<25%	3 m
970.2	10.7	8.9	3.1	2.87	F2	Y	N	N	?	1.8	>75%	–
1,034.1	33.2	18.9	6.8	2.78	F2	Y	N	N	A	14.3	<25%	–
1,035	35.2	19.5	7.0	2.79	F2	Y	N	N	?	15.7	25–50%	3 m
1,049	n.m.	n.m.	n.m.	n.d.	F4	Y	Y	Y	B	16.9	50–75%	–
1,060	20.2	n.m.	n.m.	n.d.	F?	–	–	–	–	10.4	<25%	–
1,074	35.5	n.m.	n.m.	n.d.	F?	–	–	–	–	12.6	50–75%	–

**Note:**

For an explanation of the measurements, see Methods. All figures in mm. SI, slenderness index. For explanation of wear stages and type see Methods section. MF, main wear facet; SF, side wear facet; TF, terminal wear facet; Y, present; N, not present. m, mesial; d, distal; n.m., not measurable; n.d., not determined.

Of the 90 isolated teeth which were studied, 75 are well-preserved. Six teeth are still in matrix blocks and therefore cannot be viewed from all angles. The less well-preserved teeth were damaged during or after discovery, resulting in, *e.g*., partial or entire lack of the crown.

#### Shape of isolated teeth

As can be expected, the general morphology of the isolated teeth is similar to that described for the partially articulated skull SNHM-2207-R and the tooth rows DfmMh/FV 580.1 and DfmMh/FV 896.7. Therefore, only morphological features that could not be observed in the previously described teeth are discussed here.

The lingual side of the crowns of the isolated teeth is slightly concave with a prominent, convex and expanded area of the crown extending over the longitudinal ridge along the middle. On the lingual side, there are more or less distinct apicobasal grooves paralleling the carinae. Often, the mesial groove is deeper and better defined than the distal groove.

The root is round to elliptical in cross section and tapers towards the root tip. The root has a light brown to beige color, and especially on the lower half, it shows a very fine wrinkling of the surface. The transition from root to crown is usually characterized by a somewhat darker brown band. In contrast to the other tooth regions, this area of transition has a smooth surface without wrinkles.

The asymmetry of the isolated individual teeth varies considerably. While the mesial-most dentary and premaxillary teeth are almost symmetrical, the distal dentary and maxillary teeth are very asymmetrical (Fig. 8) and show a pronounced S-shape. Upper jaw teeth show roots with a C-shaped long axis in mesiodistal view, whereas dentary teeth possess straight roots. The apex is short and shifted far to the mesial carina so that in lateral view, the tooth crowns are round to oval or rectangular in shape. This can be observed quite well in teeth DfmMh/FV 457, DfmMh/FV 478, DfmMh/FV 479, DfmMh/FV 489, and the juvenile tooth DfmMh/FV 771 (Fig. 8A). More mesial and more symmetrical teeth are DfmMh/FV 435, DfmMh/FV 436, DfmMh/FV 447, DfmMh/FV 448, DfmMh/FV 449, DfmMh/FV 450, DfmMh/FV 456, DfmMh/FV 896.7.2, and DfmMh/FV 896.7.3 (Fig. 8B). The morphology of the remaining isolated teeth is between these two extreme forms.

#### Enamel surface morphology

The enamel surface of the teeth of *Europasaurus* is wrinkled, consisting of fold-like depressions and ridges which extend approximately parallel to the long axis of the tooth and converge on the tooth apex. This wrinkling pattern most closely resembles wrinkling morphotype III described by Holwerda, Pol & Rauhut (2015) in indeterminate eusauropod teeth from the Middle Jurassic of Argentina. As also observed by Holwerda, Pol & Rauhut (2015) in their material, wrinkling does not vary with tooth position in *Europasaurus*. It does, however, vary with the ontogenic stage of the animal and the developmental stage of the teeth.

We observed that in isolated teeth of stages R3 and F1, the enamel at the tooth tip exhibits the same rough wrinkled surface, but in functional teeth, the enamel in the apical part of the crown is smooth and appears polished, consistent with other indicators of wear. The replacement teeth have smooth enamel without wrinkling in the very early ontogenetic stages R1 and R2 (DfmMh/FV 426, DfmMh/FV 492.8, DfmMh/FV 504, DfmMh/FV 578.6, DfmMh/FV 790.6, and DfmMh/FV 844.7). They possess a simple shovel-shaped crown and no root. The lingual side is flattened, and the labial side is slightly convex. The previously described raised area on the lingual side of the crown is not clearly developed yet.

#### Denticles

As noted for the *in situ* teeth of the dentigerous bones, another special feature of replacement teeth are the denticles on the mesial and distal carinae of the crown (Fig. 9, Table 4). Denticles on carinae are a plesiomorphic feature of non-sauropod sauropodomorphs (Barrett & Upchurch, 2007), and are prominent and rough in immature teeth. The further a tooth develops, the more indistinct its denticles become as a result of tooth wear. Among the isolated teeth, those classified as replacement or immature teeth (stage R1 to F1) have visible denticles, and a total of 36 of the 90 isolated teeth show denticles on their mesial carinae.

The isolated juvenile functional tooth DfmMh/FV 860 (Fig. 9C) shows only two denticles. Very indistinct denticles occur on the mesial carina of the teeth DfmMh/FV 422 and DfmMh/FV 731, as well as the teeth DfmMh/FV 460 and DfmMh/FV 707.3.2 (Fig. 9B). Denticles are lacking on the distal carina of the teeth of juvenile individuals, and are completely lacking in large and well developed teeth of adult *Europasaurus*.

#### Patterns of tooth wear

Forty-four of the isolated teeth show relatively strong facets on the crown in the form of worn and/or polished enamel (Table 4). The wear facets can be formed and advance differently.

In a very early stage of wear, no facets are visible. The smooth polish spreads from the crown tip onto the labial and lingual sides of the teeth (stage F1). The longer a tooth stays in use, the clearer the grooves and wear facets become. Normally, the terminal wear facet forms first (stage F2). This beginning wear facet has either a round or, more often, a drop-shaped appearance (Fig. 10). The round side of the drop is located on the distal side in the upper jaw and the mesial side on the lower jaw and represents the transition to the side wear facet. The pointed side of the drop thus occurs on the mesial side of the upper jaw and distal side of the lower jaw and transitions to the main wear facet.

In teeth DfmMh/FV 432, DfmMh/FV 440, DfmMh/FV 452, DfmMh/FV 516, DfmMh/FV 537, DfmMh/FV 788, DfmMh/FV 851, and DfmMh/FV 1034.1, the terminal wear facet is rotated from 10 to 50° toward the mesiolingual side (Fig. 10A). Lower jaw teeth DfmMh/FV 439, DfmMh/FV 445, DfmMh/FV 447, DfmMh/FV 456, and DfmMh/FV 896.7.3 show a weakly developed terminal wear facet, which is labiodistally inclined (Fig. 10B).

Two exceptions are DfmMh/FV 461 (Fig. 10C), where the terminal wear facet cuts obliquely onto the distal carina, and DfmMh/FV 436, which has no terminal wear facet, but a very narrow, steeply inclined facet at the distal carina and a slightly larger wear facet on the convex labial side (Fig. 10C). The unusual form of the wear facets of DfmMh/FV 461 and DfmMh/FV 436 could be due to a misalignment of the teeth in the jaw during their lifetime.

As wear progresses further, the main wear facet is usually formed very quickly (stage F3). It is a narrow, elongated facet, which extends from the crown tip to the corresponding carina (Fig. 11). The facet is always slightly concave, caused by the differential wear of the softer dentin over the harder enamel. The main wear facet is usually associated with and develops from the terminal wear facet. Exceptions are again tooth DfmMh/FV 436, which shows no terminal wear facet (Fig. 10C) and DfmMh/FV 851, which has a side wear facet instead of a main wear facet (Fig. 11C), and DfmMh/FV 439 and DfmMh/FV 896.7.3, where the main wear facet is not connected to the terminal wear facet.

As wear progresses, eventually a side wear facet forms (stage F4), but the main wear facet still dominates in size. The side wear facet proceeds deep along the carina, but it is not as wide as the main wear facet.

In rare cases, the side wear facet may eventually surpass the main wear facet in size and spread, as seen in teeth DfmMh/FV 424, DfmMh/FV 452 (Fig. 11C), and DfmMh/FV 455 (Fig. 11A). In the upper jaw teeth, DfmMh/FV 428, DfmMh/FV 454 (Fig. 11B), DfmMh/FV 472 (Fig. 11B), and DfmMh/FV 851 (Fig. 11C), only a small facet on the distal carina has formed below the terminal wear facet.

In the last stage of use, all wear facets fuse and form a joint large wear facet (stage F5), which extends over the entire width of the crown, as in the teeth DfmMh/FV 424 (Fig. 11A) and DfmMh/FV 455 (Fig. 11A). The terminal wear facet disappears as it is integrated directly into the combined main and side wear facets. The enamel band borders the entire wear facet and is raised above the dentin. Interestingly, tooth DfmMh/FV 730 shows a level wear facet with a very irregular outline. It is plausible that the crown had been broken off when the tooth was still in use, and the fracture surface was smoothed over by wear.

On some maxillary teeth with advanced wear, the main and side wear facets are not long, narrow facets along the carina, but rather incorpotated into the teardrop-shaped terminal wear facet. The pointed end of the teardrop shape is thus shifted more and more to the side of the mesial carina (Figs. 11, 12)

#### Resorption of the roots

In 47 of the isolated individual teeth, incipient or advanced resorption of the roots is apparent (Table 4). The resorption of the roots takes place more or less proportional to the wear of the teeth and is well correlated with it.

Usually, teeth in stage F1 already have fully developed roots, as seen in DfmMh/FV 433 (Fig. 13A), DfmMh/FV 488, DfmMh/FV 489 (Fig. 8A), DfmMh/FV 771 and DfmMh/FV 1060, which already has slight traces of resorption. The resorption always begins on the lingual side of the root with an oval dissolution pit of the root surface, a few millimeters above the root tip. Resorption progresses along the tooth long axis: first the root tip is resorbed, then the entire root. This is seen particularly well in the teeth DfmMh/FV 436 (Fig. 8B), DfmMh/FV 440 (Fig. 10A), DfmMh/FV 479, DfmMh/FV 486, DfmMh/FV 731 and DfmMh/FV 896.7.3 (Fig. 7B). These teeth are at stage F2, with the wear facet spreading and resorption increasing. The teeth in stage F2 usually have less than 25% of the total root length resorbed. The resorption surface on the root is slightly inclined from lingual to labial, and in rare cases, horizontal.

For the teeth at stages F3 and F4, root resorption is not always well correlated with the wear surfaces. Resorption varies between 25% and 75% of the root length. Almost all of the teeth in which over 75% of the root is resorbed, are clearly assignable to stages F4 or F5. Exceptions are tooth DfmMh/FV 435, in which the crown shows wear of stage F1, and the teeth DfmMh/FV 851 and DfmMh/FV 970.2, which also lack strong wear traces and were assigned to stage F2 or F3.

### Tooth replacement

**Figure 14:**
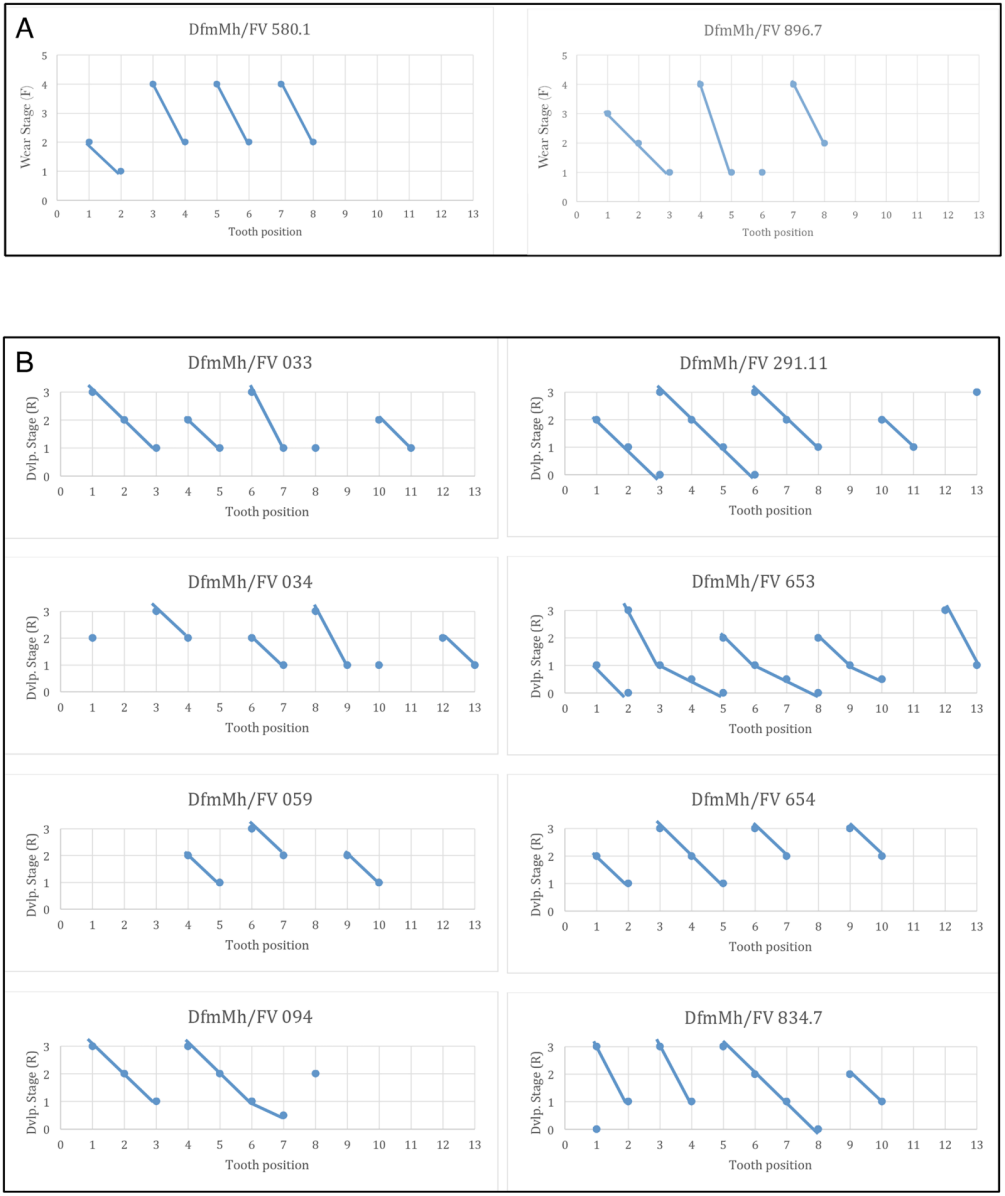
Graphs showing the different wear stages of the isolated tooth rows and dentaries of *Europasaurus holgeri* from which the Z-spacing was calculated. (A) Z-spacing of isolated tooth rows DfmMh/FV 580.1 and DfmMh/FV 896.7. (B) Z-spacing of dentaries DfmMh/FV 033, FV034, FV 059, FV 094, FV 291.11, FV 653, FV 654, and FV 834.7.

Like all sauropods, *Europasaurus* shows a well-organized tooth replacement (Figs. 2, 4–7, 14). The Z-spacing ranges between 2.5 and 3 and therefore lies within the usual sauropod values (Fig. 14). DeMar (1972) determined a Z-spacing of 1.56–2.8 for most reptiles, which may change during ontogeny. The lower the value, the higher the replacement rate (DeMar, 1972). An increase in Z-spacing is observed from the mesial to the distal tooth positions in *Europasaurus*, which is explained by the fact that the mesial dentition is more stressed and replacement occurs more frequently, thus exceeding the replacement rate in distal teeth. This is also the case for most reptiles (Edmund, 1960; Fastnacht, 2008).

The average Zahnreihe length in the dentary and ITRs of *Europasaurus* is 2 to 3 teeth (Fig. 14). Here, however, only part of the dentition is considered in each case (for the dentary, only the replacement dentition; for the ITRs, only the functional dentition). The slope of the Zahnreihe varies in the dentary from 1/2 to 2, and in the ITRs from 1 to 3. In general, however, the Zahnreihe of the functional and replacement teeth are uniform and parallel to each other.

### Ontogenetic changes in isolated teeth and dentitions

Due to the many well-preserved mandibles with replacement teeth of different developmental stages, ontogenetic changes in the teeth of *Europasaurus* can be examined in detail. Juvenile teeth are distinguished from those of adult animals by their overall smaller size. However, tooth size also decreases distally, the smallest distal teeth of adult animals are still quite robust and broadly built and show pronounced wear. Juvenile teeth, on the other hand, are much more delicate and sleek with a very narrow crown, which is only slightly wider than the root. The enamel of the crown in juvenile teeth (Fig. 9) is very thin, barely wrinkled and is similar to that of the replacement teeth.

### Assigning teeth to jaw quadrants and position in jaw

Based on observations on the tooth-bearing bones and isolated tooth rows of *Europasaurus* and on published descriptions of sauropod dentitions (Janensch, 1935–1936; Chatterjee & Zheng, 2005; Wiersma & Sander, 2017), the strong lingual curvature of the upper jaw teeth distinguished them from those of the lower jaw. Taking the middle of the crown as point of reference, the apical part of the crown and the tip of the root are curved lingually in the upper jaw teeth. In mesiodistal view, the tooth long axis of the upper jaw teeth is C-shaped (Fig. 13A). The dentary teeth, on the other hand, show less lingual curvature (Fig. 13B). Also, in the upper jaw teeth, the terminal wear facet is wider and more drop-shaped than in the teeth of the lower jaw. The terminal wear facet of the upper jaw is inclined toward the lingual side, whereas the terminal wear facet of the lower jaw is inclined to the labial side. These features in combination allow a distinction between upper and lower teeth.

In labial or lingual view, the tooth crown shows a variably pronounced asymmetry along the mesial carina, resulting in an S-shape (Fig. 8), as also described for *Bellusaurus* (Moore et al., 2018). The apex of the tooth is shifted towards distal, as is the crown base. The middle part of the crown is shifted toward mesial. The bulge on the labial side of the crown appears slightly curved with the convex side directed mesially and is always located on the mesial side (Fig. 8). This allows for a distinction between left and right teeth.

To differentiate between mesial and distal teeth, the distally increasing asymmetry can be used (Chatterjee & Zheng, 2005; Kosch, 2014; Kosch et al., 2014; this study).

## Discussion

### Early stage and location of tooth formation

In the dentigerous bones of *Europasaurus* (Figs. 2–4), we observed in lingual and occlusal view that the replacement teeth initially appear to form in a crypt spatially separate from the alveolus of the functional tooth. The functional teeth are generally lost, and the alveoli are empty or contain older replacement teeth. Initial formation of the replacement teeth in a crypt happens deep within the alveolar ramus.

### Comparison with other sauropod dentitions

The teeth and dentition of *Europasaurus* have the same attributes as in other basal Macronaria (Janensch, 1935–1936; White, 1958; Madsen, McIntosh & Berman, 1995; Chatterjee & Zheng, 2005; Chure et al., 2010). There thus are no morphological changes that could be directly linked to evolutionary dwarfing. Comparison with the dentition of two closely related taxa, *Camarasaurus* and *Giraffatitan*, reveals clear similarities to *Europasaurus*. The dental formula of *Europasaurus* (Marpmann et al., 2015) is similar to that of *Giraffatitan* with pm4 + m11–13/d12–14 (Janensch, 1935–1936; Christiansen, 2000). *Camarasaurus* hast a dental formula of pm4 + m8–10/d11–14 (Janensch, 1935–1936; Madsen, McIntosh & Berman, 1995; McIntosh et al., 1996; Christiansen, 2000; Chatterjee & Zheng, 2005; Wiersma & Sander, 2017) and thus the same number of teeth in the dentary and premaxilla as *Europasaurus* and *Giraffatitan*, but the number of maxillary teeth is lower. The Z-spacing of all three sauropods have values between 2.0 and 3.0 (Chatterjee & Zheng, 2005; Kosch, 2014; Kosch et al., 2014; this study).

Morphologically, the teeth of *Europasaurus* are sleeker in appearance than those of *Camarasaurus*, but are slightly more robust and more closely set than those of *Giraffatitan*. The SI ranges from 1.0 to 2.0 in *Camarasaurus* (Wiersma & Sander, 2017) but from 2.5 to 3.0 in *Giraffatitan* (Chure et al., 2010) and *Europasaurus* (this study), where *Europasaurus* scores mainly in the lower range. *Europasaurus* thus falls in the “broad-crowned” category of teeth (Upchurch, 1998; Upchurch & Barrett, 2005; Chure et al., 2010). The teeth of *Camarasaurus* are labiolingually flattened and mesiodistally widened (Wiersma & Sander, 2017), more so than in *Europasaurus*. In *Camarasaurus*, the rounded ridge on the lingual side is very pronounced and the grooves, paralleling the carinae, are present on both the labial and the lingual side. The teeth are only slightly bent lingually and have an increasing asymmetry towards the distal end of the tooth row.

The teeth of *Giraffatitan* exhibit a marked asymmetry, which increases posteriorly (Janensch, 1935–1936; V. Régent, 2011, personal observation). They are strongly curved lingually. The lingual side of the tooth crown is concave, and the labial side is convex, resulting in a D-shaped tooth cross section. The labial grooves are often only pronounced along the distal carina. Lingually, a groove is evident along the mesial carina (Janensch, 1935–1936; V. Régent, 2011, personal observation). *Camarasaurus* is not reported to have denticles on either the replacement teeth or the functional teeth (Upchurch & Barrett, 2000; Chatterjee & Zheng, 2005; Wiersma & Sander, 2017), whereas three teeth of *Giraffatitan* bear indistinct denticles (Janensch, 1935–1936; Upchurch & Barrett, 2000). *Europasaurus* shows distinct and coarse denticles on immature tooth crowns. These denticles are worn off in the functional teeth. The ‘en echelon’ pattern of the teeth observed in *Europasaurus* is a synapomorphy of Eusauropods and has been described in the other sauropods detailed above as well as in others (Wilson & Sereno, 1998; Chatterjee & Zheng, 2005; Sereno & Wilson, 2005; Carballido et al., 2017).

The intermediate morphology of the *Europasaurus* tooth morphology between *Giraffatitan* and *Camarasaurus* thus falls in line with its intermediate skeletal morphology. The intermediate morphology has made it difficult to determine the phylogenetic position of *Europasaurus*, either as a basal macronarian more derived than *Camarasaurus* or as a member of Brachiosauridae (Carballido & Sander, 2014; Carballido et al., 2019). Dwarfing may have resulted in stem-ward slippage of the taxon (Carballido et al., 2019). Further research in this matter is warranted, for example the interpretation of the conspicuous denticles in *Europasaurus* replacement teeth in a phylogenetic context (J. Whitlock, 2023, personal communication).

### Tooth wear and food intake

**Figure 15:**
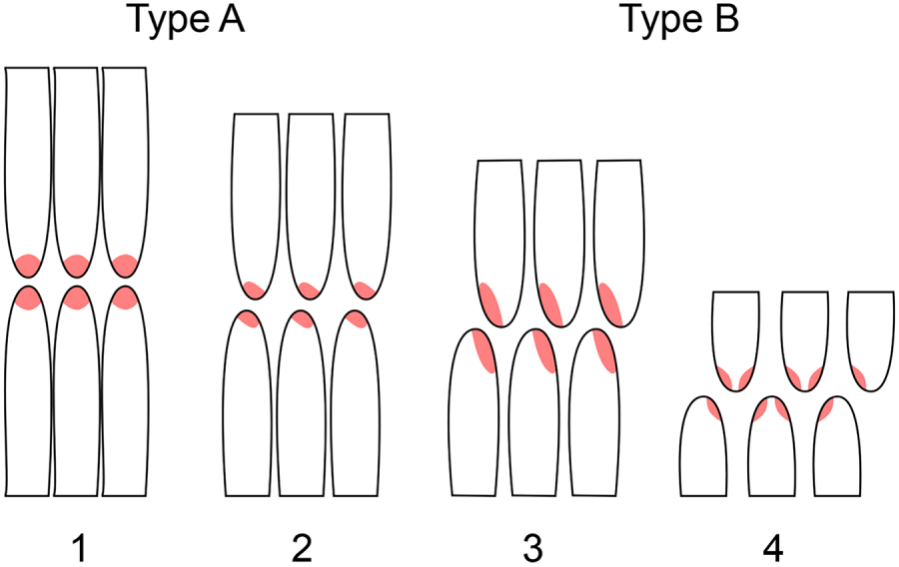
Schematic representation of the two wear types and four subtypes of sauropod dentitions. Often, two or three of these types occur in a single individual or even on the same tooth. *Europasaurus* shows wear of both: type A (subtype 2), as well as type B (subtypes 3, 4). *Camarasaurus* possesses only type B, and *Giraffatitan* mainly type B (subtype 3). Modified from Saegusa & Tomida (2011) and Wiersma & Sander (2017).

There are two different types of wear in sauropod teeth, first described by Calvo (1994), that also occur in the upper jaw teeth of *Europasaurus*. Type A wear results in a flat, smooth wear facet (*e.g*., Figs. 1, 9A, 9B and 10A), which does not, or only very late in wear, encroaches on the carinae (Fig. 15). Here, contrary to most wear facets, the enamel always forms a level surface with the dentin, *i.e*., the dentin is not eroded out relative to the enamel. In type B of Calvo (1994), however, wear reaches the mesial and distal carinae early, and the facet is V-shaped (Fig. 15). The enamel always is raised above the softer dentin. Type A appears along the entire tooth row but the facet becomes increasingly asymmetrical in the smaller distal teeth, whereas type B primarily occurs in the mesial teeth of *Europasaurus*.

These two types are less pronounced in the dentary teeth which generally show less wear than the maxillary teeth. Often, only the tip of the crown is affected by wear. Mesial teeth usually have a horizontal terminal wear facet, but both main and side wear facets occur on the carinae. The more asymmetrical the teeth are, the more asymmetrical are the terminal wear facets, being occasionally strongly shifted to the distal carina, for example in teeth DfmMh/FV 445 and DfmMh/FV 461.

It is a special feature of *Europasaurus* that both types of Calvo’s wear facets are developed (Fig. 15; Table 4). Type A, expressed in *Europasaurus* as tear-drop shaped terminal wear facets, is subordinately observed in *Giraffatitan* (Janensch, 1935–1936) and is caused by tooth-to-tooth contact (Greaves, 1973), but also caused by biting on hard and tough food (Janensch, 1935–1936; Upchurch & Barrett, 2000; Grippo, Simring & Schreiner, 2004). This facet is exclusively limited to the top of the crown. The teeth located more posteriorly have more asymmetric facets. This suggests that the teeth of *Europasaurus* did not interlock, unlike those of *Camarasaurus* (Calvo, 1994; Upchurch & Barrett, 2000; Chatterjee & Zheng, 2005; Wiersma & Sander, 2017), but rather that the lingual side of an upper jaw tooth met the labial side of its antagonist in the lower jaw (Figs. 1, 10).

Type B, the V-shaped wear facet, occurs mainly in the mesial teeth of *Europasaurus*. Type B, subtype (Fig. 15), wear facets are the most common in *Giraffatitan* as well (V. Régent, personal observation; Kosch et al., 2014). These wear facets probably were not caused by tooth-to-tooth contact, but primarily by stripping and snipping off vegetation (Christiansen, 2000). The basal extent of the wear facets along the carinae indicates that they could not have possibly been produced by antagonists, unless there was an interlocking organization of the upper and lower dentition. Arguing against such wear caused by interlocking of teeth is the dense spacing of the teeth in the ITRs, however. On the other hand, in these facets, the enamel band always stands proud of the exposed dentin, which is typical of wear caused by foodstuffs (Grippo, Simring & Schreiner, 2004).

It is plausible that *Europasaurus* consumed different types of foodstuffs. Its relatively robust and broad-crowned teeth were adapted to snap off small twigs and consume tough vegetation, as in other broad-crowned sauropods (Sander et al., 2010; Gee, 2011; Hummel & Clauss, 2011). The mesial and distal wear facets suggest that *Europasaurus* also used its dentition like a rake, as also was hypothesized for other sauropods (Christiansen, 2000; Upchurch & Barrett, 2000; Barrett & Upchurch, 2005; Hummel & Clauss, 2011). *Europasaurus* probably did not perform branch stripping in the narrow sense because it does not show the adaptations that have been hypothesized for this feeding mode, which has been drawn into question even for diplodocid sauropods (Whitlock, 2017).

Microwear studies on the teeth of *Camarasaurus* showed that a ‘chewing-like’, transverse movement of the jaw took place upon closure (Calvo, 1994; Christiansen, 2000). This lateromedial movement of the jaw would also be an explanation for the drop-shaped wear facet of type A observed in *Europasaurus* and may indicate that the food was at least partially processed orally, as suggested by some authors for *Camarasaurus* (Calvo, 1994; Christiansen, 2000; Upchurch & Barrett, 2000).

### Isolated tooth rows as evidence for anchoring soft tissue structure

Isolated yet still articulated tooth rows are a peculiar but widespread feature among Sauropoda, not only of *Europasaurus*, and the anatomical structure that led to their preservation may be an autapomorphy of the group. ITRs have been found in *Shunosaurus* (Chatterjee & Zheng, 2002), *Abydosaurus* (Chure et al., 2010), *Giraffatitan* (Janensch, 1935–1936; Kosch et al., 2014), diplodocoids (Britt, Scheetz & Dangerfield, 2008; Whitlock, Wilson & Lamanna, 2010; Peterson et al., 2022), and titanosaurs (*Phuwiangosaurus*, V. Suteethorn, 2015, personal communication). Notably, ITRs have not been reported for *Camarasaurus* to our knowledge, despite the abundance of this taxon in the Morrison Formation. The interpretation of this peculiar kind of ITR preservation is potentially relevant to anatomy and feeding behavior of sauropods. The partially articulated *Europasaurus* skull SNHM-2207 provides evidence that ITRs of sauropods cannot in all cases be attributed to jaw bone loss due to decay, with only the teeth remaining as fossils (McIntosh, 1981), or to consumption of dentigerous bones by insects (Britt, Scheetz & Dangerfield, 2008). In fact, the specific position and preservation of the isolated upper and lower tooth rows in SNHM-2207 provides strong evidence that the preferential destruction of jaw bones is not a plausible explanation for ITRs at all.

A novel hypothesis as to the nature of the ITRs was recently advanced by Peterson et al. (2022) based on a specimen of *Apatosaurus* from the Morrison Formation of Wyoming, USA. However, the ITR in question is only represented as a cast in the study of Peterson et al. (2022). Based on this single ITR specimen, these authors hypothesize that sauropods replaced their teeth as entire rows at once. They did not consider other specimens of ITRs, and the evidence from the cast is different from what we report here and what has been described in the other ITRs before (Chatterjee & Zheng, 2002; Chure et al., 2010; Janensch, 1935–1936; Britt, Scheetz & Dangerfield, 2008; Whitlock, Wilson & Lamanna, 2010). Except for Chure et al. (2010), these studies are not cited by Peterson et al. (2022). The peculiar and confusing feature in the ITR cast in Fig. 14 of Peterson et al. (2022) is that the unworn teeth protrude well beyond the worn teeth, making it difficult to understand how tooth wear was incurred in this ITR, and raises concerns that the ITR was incorrectly restored during preparation.

Schäfer (1972) provides taphonomic observations on extant porpoises that may shed light on the issue. Mummification of porpoise cadavers causes intensive dehydration and shrinkage of the gingival connective tissue and periosteum. Shrinkage pulls the teeth from the alveoli but preserves the dentition in its original configuration in the dessicated gum tissue (Schäfer, 1972). If this observation is applied to *Europasaurus* and other Eusauropoda, it would suggest the presence of some kind of connective tissue that kept the teeth together even after death.

#### Evidence from Europasaurus and Camarasaurus

The position of the teeth in the partially articulated skull SNHM-2207-R and the ITRs DfmMh/FV 580.1 and DfmMh/FV 896.7 suggests either special taphonomic circumstances (mummification prior to burial) or, more plausible due to similar sauropod ITRs found from different depositional and taphonomic settings, the presence of some kind of strong, connective tissue holding the teeth in place, *i.e*., in a rhamphotheca-like structure, during decay and disintegration of the carcasses.

The hypothesis of a rhamphotheca-like structure is supported by the observation that in some instances, tooth roots were so strongly resorbed that the respective tooth was no longer anchored in the jaw bone, but is still found in it correct anatomical position in the tooth row. This is seen in the ITR DfmMh/FV 580.1 of *Europasaurus*.

Further evidence for such a rhamphotheca-like structure is provided by the pattern of enamel surface wear and wrinkling in the dentition of *Europasaurus*, where the wrinkled surface is worn smooth in the apical part of the crown but not in its basal part. This observation has also been reported for *Camarasaurus* sp. SMA 0002 (Wiersma & Sander, 2017). In fact, we suggest that the wrinkled surface of sauropod enamel may have evolved for improving the anchorage of the rhamphotheca-like structure on the teeth because earlier-branching sauropodomorphs have smooth enamel. Detailed study of the wrinkling of *Europasaurus* tooth enamel would clearly be rewarding but is beyond the scope of this article. In our study, it is the distribution and wear patterns on the wrinkled surface in the tooth life cycle that are of interest, not so much the fine details of wrinkle morphology.

We note that the distribution of tooth surface wear obliterating the wrinkling coincides with the maximum basal extent of the wear facets (*e.g*., Fig. 1). Even in the most worn teeth, wear does not affect the entire crown but only somewhat more than its apical half, and the wrinkling would have aided in keeping the tooth in place in the rhamphotheca until it was finally shed.

Evidence for a rhamphotheca-like structure is also provided by the jaws of *Camarasaurus* sp. SMA 0002 (Wiersma & Sander, 2017), in form of soft part preservation. A patch of what appears to be fossilized skin covers more than 50% of the crowns on the left lower jaw of the specimen. This patch is a 100 × 100 mm-sized, thin layer of a skin-like structure exposed during preparation. It covers the tooth necks and most of the tooth crowns of the 5th to 8th left dentary teeth (Wiersma & Sander, 2017). A similar but somewhat smaller structure is also found in the area of the 1st and 2nd left dentary teeth of SMA 0002 (Wiersma & Sander, 2017). Skin preservation appears to be common in the Howe Ranch dinosaur quarries (see Tschopp, Mehling & Norell, 2020).

A striking feature of both *Camarasaurus* (specimen SMA 0002) (Wiersma & Sander, 2017) as well as *Giraffatitan* (MB.R.2223 = t1) is that the functional teeth protrude far out of the jaw, and the tooth necks are exposed. In neither specimen can this feature be attributed to taphonomy. This is further suggestive of the existence of a strong, connective tissue structure within Macronaria, completely covering the sensitive and unprotected tooth necks and providing additional anchorage for the dentition. It has been suggested that eusauropods in general possessed a structure akin to the rhamphotheca in birds and some non-avian theropods (Wiersma & Sander, 2017), in which the teeth were deeply embedded, only exposing parts of their crown (Wiersma & Sander, 2017). This rhamphotheca-like structure together with the teeth would have formed an efficient cropping apparatus adapted to fast wear caused by a high food intake (Sander, 2013; Wiersma & Sander, 2017). This hypothesis is consistent with the extremely rapid tooth replacement documented for some sauropods (D’Emic et al., 2013).

#### Keratinous oral structures in dinosaurs

Keratinous structures on plesiomorphically dentigerous bones, *i.e*., the premaxilla, maxilla, and dentary, are not unusual in dinosaurs and have evolved independently in different clades of Dinosauria. A rhamphotheca is considered synapomorphic for Ornithischia and evolved many times in Saurischia (Louchard & Viriot, 2011; Wang et al., 2017; Wang, O’Connor & Zhou, 2019; Button et al., 2023). In early ornithischians, such as *Heterodontosaurus* and probably *Kulindadromeus*, there is a rhamphotheca combined with teeth on the premaxilla (Sereno, 2012; Godefroit et al., 2014).

Among theropods, the phylogenetically and temporally earliest occurrence is the ceratosaur *Limusaurus* from the Middle Jurassic of China (Wang et al., 2017) that, in addition to ontogenetic tooth loss, combines teeth and a rhamphotheca on the same jaw bone. Similarly, the therizinosaur *Erlikosaurus* may have combined a rhamphotheca with a dentigerous maxilla and dentary (Button et al., 2023). A third case of at least a close developmental proximity of a beak and teeth was described by Wang, O’Connor & Zhou (2019) in a caenegnathid oviraptorosaur from China in which teeth also were lost ontogenetically and their place was taken by a rhamphotheca. The pattern of multiple instances of beak evolution and tooth loss in Mesozoic birds was reviewed by Louchard & Viriot (2011). However, these authors did not address the co-occurence of teeth and a beak in any detail, citing the need for further research, concluding that “There is a need to investigate the presence and extent of the rhamphotheca in different lineages of Mesozoic birds, and the evolution of its spatial relation with dentition”.

Among sauropods, and apart from the study on *Camarasaurus* by Wiersma & Sander (2017), there have only been two instances where a keratinous jaw structure has been reconstructed for a sauropod. One is the titanosaur *Bonitasaura*, for which Apesteguía (2004) and Gallina & Apesteguía (2011) reconstructed a keratinous cutting edge posterior to the tooth row on the dentary and called it a “beak-like structure” (Apesteguía, 2004). The other is the brief mention of a post-dental keratinous structure in *Nigersaurus* by Sereno et al. (2007).

The description of a “beak-like structure” by, *e.g*., Apesteguía (2004) is somewhat misleading and has led perhaps to false perceptions (Button, 2018, p. 2). A beak or rhamphotheca is a structure covering the tip of the jaw and extending caudally to varying degrees (Button, 2018). In *Bonitasaura*, the hypothesized keratinous cutting structures are caudal to the dentary teeth (Apesteguía, 2004; Gallina & Apesteguía, 2011) and thus probably not homologous (contra Button, 2018) to the rhamphotheca of theropods and to he structure we hypothesize here. We also do not subscribe to the argument of Button (2018, p. 83) that “the rhamphotheca is a functional replacement for dentition” and hence “there is no reason to have both a beak and teeth on the same element in the same location of the jaw.” There is no *a priori* reason why teeth and a rhamphotheca should exclude each other.

Perhaps our study shows that the classical assessment, as summed up by Button (2018) above, is not necessarily true. Given the extreme bauplan of sauropods, it may come as no surprise that they show unique feeding adaptations, including a rhamphoteca-like structure supporting the action of the teeth. Our hypothesis thus may integrate well with the unusual pattern and extremely high rate of sauropod tooth replacement (D’Emic et al., 2013). Alternatively, another kind of cornified connective tissue, not homologous to a keratinous rhamphotheca, could be envisaged to explain the pattern we observe. Clearly, further detailed study of the anatomy, microanatomy, and histology of the dentigerous bones in sauropods is needed.

## Conclusions

*Europasaurus* fossils include superb dental and jaw material, consisting of numerous isolated jaw bones, a partial skull with an ITR in the process of separation, four ITRs (of which two could be studied), and numerous isolated teeth. In addition, dentigerous bones, especially the dentary, and teeth are represented by ontogenetic series. The dental formula (pm4 + m12/d13–14) matches that of *Giraffatitan brancai* (Janensch, 1935–1936), and the two species share similar tooth morphologies, suggesting close relationships between *Europasaurus* and the Brachiosauridae. *Europasaurus* has typical basal macronarian teeth, including a shovel-like shape and lingually curved and mesiodistally enlarged teeth. The SI of the teeth is between 2.5 and 3, indicating ‘broad-crowned’ teeth. The teeth increase in asymmetry and decrease in size from mesial to the distal. Dental features such as the degree of convexity of the mesial carinae, curved roots in the upper jaw teeth, and asymmetry can be used to assign isolated teeth to an approximate position in the jaw. The isolated teeth, the ITRs, and articulated teeth in the jaws of *Europasaurus* possess typical developmental and functional patterns controlled by the ontogeny of the individual, the developmental stage of the tooth, and the position of the tooth in the jaw. Regarding the denticles, three patterns are apparent: (1) The replacement teeth of juvenile individuals have better developed denticles than adult replacement teeth, a case of recapitulation. (2) Distal replacement teeth in the same jaw have better developed denticles than mesial teeth. (3) Denticles are lost through wear. Two types of facets results from wear. For one, there are flat terminal ones, lacking differential removal of the dentin, and second, there are mesial and distal ones with preferential abrasion of the dentin and raised enamel edges. Wear facets increase with use of the tooth, and at the same time, the root of the tooth becomes resorbed until it is finally replaced by a new tooth. In total, four developmental and five wear stages can be observed in *Europasaurus* teeth.

Effects of dwarfism on the dentition itself cannot be detected. *Europasaurus* has absolutely smaller teeth than most other sauropods, but all proportions and relevant characteristics are retained. Only the presence of denticles differs from the other derived sauropods and could therefore be directly related to dwarfism by representing a plesiomorphic feature resulting from paedomorphosis.

The partially articulated skull SNHM-2207-R and the ITRs DfmMh/FV 580.1 and DfmMh/FV 896.7 suggest the presence of some kind of strong, connective tissue holding the teeth in place in a rhamphotheca-like structure. We hypothesize that eusauropods in general may have possessed a structure akin to a rhamphotheca, which may be an autapomorphy of the group.

The enormous size difference between the very large *Giraffatitan* and the diminutive *Europasaurus* raises important questions as to differences in diet and feeding styles between the two species as well as other, normal-sized members of the clade. Diet and feeding style of *Europasaurus* would certainly have been constrained by its island habitat, of which we know relatively little. Also, the allometry of dwarfing surely will be important, but it remains largely unstudied in *Europasaurus*. Clearly, there is much fertile ground for future work in these areas.

## Repository abbreviations

DfmMh/FV: Dinosaurier-Freilichtmuseum Münchehagen/Verein zur Förderung der niedersächsischen Paläontologie, Münchehagen, Germany
MfN: Museum für Naturkunde, Berlin, Germany
SMA: Sauriermuseum Aathal, Aathal Switzerland
NLMH: Niedersächsisches Landesmuseum, Hannover, Germany
SNHM: Staatliches Naturhistorisches Museum, Braunschweig, Germany

## Additional Information and Declarations

### Data Availability

The following information was supplied regarding data availability: The specimens are reposited as follows: Skull: NLMH 105.996; SNHM 2207-R. Premaxillae: DfmMh/FV 032, 061, 652.2, 982, 291.18. Maxillae: DfmMh/FV 291.17. Dentaries: DfmMh/FV 033, 034, 059, 092, 093, 094, 290, 291.11, 501, 653, 654, 834.7. 1058.14. ITRs: DfmMh/FV 580.1, 896.7. Isolated teeth: DfmMh/FV 278, 280, 422, 424, 426, 428, 429, 430, 431, 432, 433, 434, 435, 436, 437, 438, 439, 440, 441, 442, 443, 444, 445, 446, 447, 448, 449, 450, 451, 452, 453, 454, 455, 456, 457, 458, 459, 460, 461, 462, 472, 478, 479, 486, 487, 488, 489, 492.8, 495.6, 496, 504, 516, 537, 558, 578.6, 607, 636, 660 662, 663.1, 707.3.1, 707.3.2, 726, 727, 730, 731, 771, 788, 790.6, 790.7, 844.7, 848, 851, 860, 865.1, 868.2, 869, 876, 888, 899, 896.7.2, 896.7.3, 948, 967, 970.2, 1034.1, 1035, 1049, 1060, 1074. Skeletons: MfN MB.R.2180, MB.R.2181, MB.R.2223, MB.R. 2390; SMA, 0002, 0004,0011.
